# Global DNA Hypomethylation in Epithelial Ovarian Cancer: Passive Demethylation and Association with Genomic Instability

**DOI:** 10.3390/cancers12030764

**Published:** 2020-03-24

**Authors:** Wa Zhang, David Klinkebiel, Carter J. Barger, Sanjit Pandey, Chittibabu Guda, Austin Miller, Stacey N. Akers, Kunle Odunsi, Adam R. Karpf

**Affiliations:** 1 Eppley Institute for Research in Cancer, University of Nebraska Medical Center, Omaha, NE 68198, USA; wa.zhang@beigene.com (W.Z.); Carter.Barger@ucsf.edu (C.J.B.); 2 Fred & Pamela Buffett Cancer Center, University of Nebraska Medical Center, Omaha, NE 68198, USA; dklinkebiel@unmc.edu (D.K.); babu.guda@unmc.edu (C.G.); 3 Department of Biochemistry and Molecular Biology, University of Nebraska Medical Center, Omaha, NE 68198, USA; 4 Department of Genetics, Cell Biology, and Anatomy, University of Nebraska Medical Center, Omaha, NE 68198, USA; sanjit.pandey@unmc.edu; 5 Department of Biostatistics, Roswell Park Comprehensive Cancer Center, Buffalo, NY 14263, USA; austin.miller@roswellpark.org; 6 Department of Gynecologic Oncology, Roswell Park Comprehensive Cancer Center, Buffalo, NY 14263, USA; stacey.akers@roswellpark.org (S.N.A.); kunle.odunsi@roswellpark.org (K.O.); 7 Department of Immunology, Roswell Park Comprehensive Cancer Center, Buffalo, NY 14263, USA; 8 Center for Immunotherapy, Roswell Park Comprehensive Cancer Center, Buffalo, NY 14263, USA

**Keywords:** DNA hypomethylation, epithelial ovarian cancer, gene expression, repetitive elements, genomic instability, DNMTs

## Abstract

A hallmark of human cancer is global DNA hypomethylation (GDHO), but the mechanisms accounting for this defect and its pathological consequences have not been investigated in human epithelial ovarian cancer (EOC). In EOC, GDHO was associated with advanced disease and reduced overall and disease-free survival. GDHO (+) EOC tumors displayed a proliferative gene expression signature, including *FOXM1* and *CCNE1* overexpression. Furthermore, DNA hypomethylation in these tumors was enriched within genomic blocks (hypomethylated blocks) that overlapped late-replicating regions, lamina-associated domains, PRC2 binding sites, and the H3K27me3 histone mark. Increased proliferation coupled with hypomethylated blocks at late-replicating regions suggests a passive hypomethylation mechanism. This hypothesis was further supported by our observation that cytosine DNA methyltransferases (*DNMTs*) and *UHRF1* showed significantly reduced expression in GDHO (+) EOC after normalization to canonical proliferation markers, including *MKI67*. Finally, GDHO (+) EOC tumors had elevated chromosomal instability (CIN), and copy number alterations (CNA) were enriched at the DNA hypomethylated blocks. Together, these findings implicate a passive DNA demethylation mechanism in ovarian cancer that is associated with genomic instability and poor prognosis.

## 1. Introduction

Altered DNA methylation, a fundamental characteristic of human cancer, includes gains and losses of methylation [1,2]. DNA hypermethylation leads to tumor suppressor gene silencing and occurs frequently at genomic regions occupied by polycomb group proteins in embryonic stem cells [1,3]. In contrast, DNA hypomethylation is “global,” as 5-methyl-deoxycytidine (5mdC) levels are often reduced in cancer [4,5,6]. In agreement, DNA hypomethylation occurs at repetitive elements (RE), including the interspersed retrotransposon *LINE-1* [7], which accounts for ~17% of the genome. Global DNA hypomethylation (GDHO) is also associated with hypomethylation and activation of cancer-testis or cancer-germline (CG) genes [8,9,10,11,12,13]. Epigenomic approaches have revealed that GDHO is not random or driven solely by changes at RE, but rather is localized to large genomic regions referred to as hypomethylated blocks [14,15,16]. Hypomethylated blocks overlap lamina-associated domains (LADs) and, interestingly, can contain epigenetically silenced genes, as well as genomic regions showing high gene expression variability [15,17,18]. 

GDHO in cancer tissues and cells is commonly determined using RE methylation as a biomarker, including, most frequently, *LINE-1* methylation. In addition, GDHO is often associated with poor prognosis, but the reasons for this association are not well established [19]. Several plausible mechanisms may account for this link. First, GDHO may promote chromosomal instability (CIN), as genetically induced DNA hypomethylation in mouse tumor models and human cancer cell lines causes aneuploidy, chromosomal translocations, and copy number alterations (CNA) [20,21,22]. Supporting this idea, DNA hypomethylation and genomic alterations are associated in human cancer [23,24,25,26,27,28]. Second, aberrant gene expression, including oncogene activation, RE expression, or CG antigen gene activation may promote oncogenic phenotypes and/or disease progression [2,29,30,31,32]. Third, GDHO and the associated hypomethylated block formation may promote gene expression variability and provide a selective growth advantage to the effected cancer cells [33].

In addition to identifying the genomic targets and biological consequences of GDHO, it is important to also understand its origin. Two general mechanisms might underlie GDHO. First, active hypomethylation, caused by a molecular alteration that disrupts DNA methylation or enhances DNA demethylation, could be involved. Potential active mechanisms would include mutations in *DNMTs* or *Ten-eleven translocation methylcytosine dioxygenase* (*TET*) genes. Second, passive hypomethylation could occur. Passive hypomethylation emanates from the fact that DNA methylation is a post-replicative DNA modification that could, under certain circumstances, become unlinked from DNA replication. In the passive model, hypomethylation was proposed to indirectly result from cellular transformation and the accompanied increase in cell proliferation [34]. Notably, a recent seminal study demonstrated a link between DNA hypomethylation, late replication timing, and mitotic cell division in several different human cancers [28]. Despite this work, little information is currently available on what drives DNA hypomethylation in the context of EOC [9].

RNA sequencing (RNA-seq) is commonly used to study promoter variation and splice variants, which are difficult to measure using microarrays [35]. Moreover, RNA-seq experiments can be designed to measure RE-derived transcripts, which have traditionally been omitted from studies of the cancer transcriptome. In fact, total RNA-seq data revealed frequent and widespread expression of RE in pancreatic cancers and other tumors [36]. The mechanisms accounting for RE expression in cancer are largely unknown, but potentially include epigenetic activation by DNA hypomethylation. 

Epithelial ovarian cancer (EOC) and its most common subtype, high-grade serous ovarian cancer (HGSOC), is the most lethal gynecologic malignancy [37]. EOC is characterized by widespread CNA, *TP53* mutations, defects in homologous recombination (HR), retinoblastoma protein (RB) pathway dysregulation, *CCNE1* amplification, and FOXM1 pathway activation [38]. In addition to these genetic changes, EOC shows altered DNA methylation, including both hyper- and hypomethylation [39]. More specifically, GDHO, including *LINE-1* hypomethylation, is a common phenotype observed in EOC tissues [9,40,41]. *LINE-1* hypomethylation and expression is also common in ovarian cancer precursor lesions known as serous tubal intraepithelial carcinomas (STICs) [42]. Here, we studied the phenomenon of GDHO in EOC, including its clinico-pathological context, its molecular underpinnings, and its relationship to CIN. 

## 2. Results

### 2.1. LINE-1 Hypomethylation is Associated with Disease Progression and Reduced Survival in EOC 

**Figure 1:**
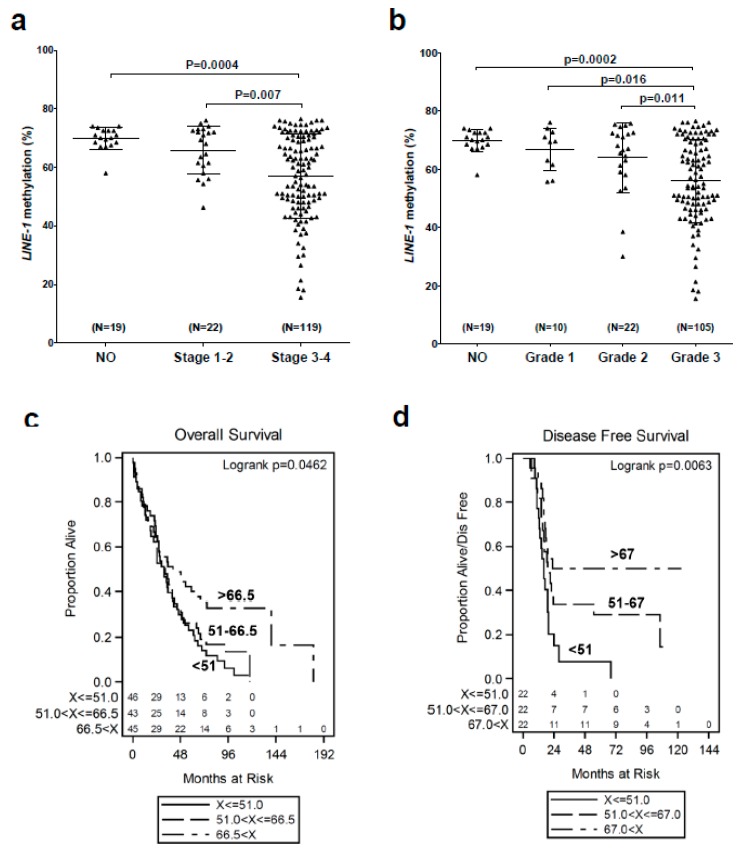
*LINE-1* hypomethylation is associated with advanced disease and reduced survival in EOC. *LINE-1* methylation was determined by sodium bisulfite pyrosequencing. (**a**) *LINE-1* methylation vs. clinical stage. (**b**) *LINE-1* methylation vs. pathological grade. For **a** and **b**, mean ± SD is plotted, and Mann–Whitney p-values are indicated. (**c**,**d**) Kaplan–Meier survival analyses and log rank test p-values of EOC patients separated based on tumor *LINE-1* methylation values. (**c**) *LINE-1* methylation vs. overall survival. Patients were separated into three groups based on *LINE-1* methylation values: low (<51.0%), middle (51.0–66.5%), and high (>66.5%). A key for the three groups, and the number of patients in each, is shown. (**d**) *LINE-1* methylation vs. disease-free survival. Patients were separated into three groups based on *LINE-1* methylation values: low (<51.0%), middle (51.0–67.0%), and high (>67.0%). A key for the three groups, and the number of patients in each, is shown.

Previously, we validated *LINE-1* methylation as a biomarker of global DNA methylation in EOC, and reported that *LINE-1* is hypomethylated in EOC when compared to normal ovary (NO), ovarian surface epithelia (OSE), and fallopian tube epithelia (FTE) tissues [9,43]. Here, we assessed the relationship between *LINE-1* methylation and EOC clinico-pathology. *LINE-1* hypomethylation increased with advanced clinical stage and histopathological grade, and correlated with reduced overall and disease-free survival (Figure 1a–d). Furthermore, the association of *LINE-1* hypomethylation with disease-free survival remained significant in a proportional hazards model, after adjustment for age (HR *p* = 0.03). These data thus show that GDHO is linked to advanced disease and poor prognosis in EOC.

### 2.2. GDHO (+) EOC Tumors have Distinct Patterns of Gene Expression, including Enriched Signatures for Cell Proliferation 

**Figure 2:**
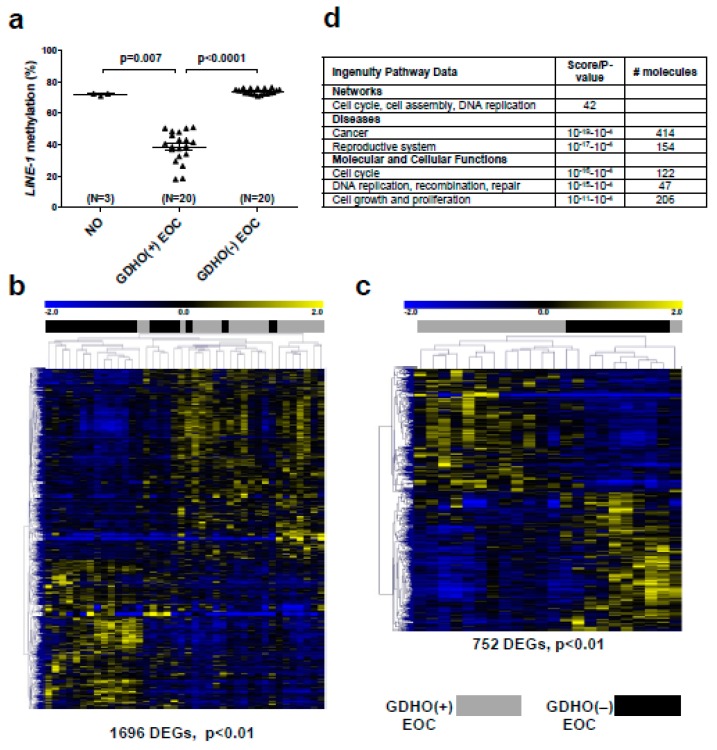
GDHO (+) EOC show distinct patterns of gene expression. (**a**) *LINE-1* methylation in sample groups used for Affymetrix gene expression analyses: bulk normal ovary (NO), GDHO (+) EOC (i.e., *LINE-1* hypomethylated group), GDHO (–) EOC (i.e., *LINE-1* hypermethylated group). (**b**) Hierarchical clustering heat map of genes differentially expressed (p<0.01) between GDHO (+) vs. GDHO (–) EOC (all samples). (**c**) Hierarchical clustering heat map of genes differentially expressed between disease-matched (age-matched, stage 3/4, grade 3, serous EOC) GDHO (+) vs. GDHO (–) EOC. All tumors correspond to the HGSOC subtype. The number of genes showing significant differential expression in each comparison is indicated. Sample identities are indicated at top (see key). (**d**) Selected Ingenuity Pathway Analyses (IPA) data for the GDHO (+) vs. GDHO (–) EOC microarray comparison.

We used gene expression microarrays to profile: (1) EOC showing significant *LINE-1* hypomethylation (i.e., GDHO (+) EOC; N = 20), (2) EOC with *LINE-1* methylation levels similar to NO (i.e., GDHO (–) EOC; N = 20), and 3) NO; N = 3 (Figure 2a). We used NO as a control because we had difficulty obtaining high quality RNA from primary OSE and FTE tissues. Hierarchical clustering of differentially expressed genes (DEG) revealed distinct patterns of gene expression in GDHO (+) vs. GDHO (–) EOC (Figure 2b). Using a cutoff of *p* < 0.01, 1696 Affymetrix probe sets (genes) were differentially expressed in the two groups, with 958 (56%) of these up-regulated in GDHO (+) EOC (Figure 2b; Table S1). Clustering of DEG using a false discovery rate (FDR) cutoff of 0.1 resulted in a similar separation of GDHO (+) from GDHO (−) samples (Figure S1a). Because GDHO is associated with disease progression (Figure 1), we next conducted sub-group analysis using disease-matched tumors, in which we compared age-matched, stage 3/4, grade 3, serous EOC (i.e., HGSOC) samples from each group (GDHO (+) N = 13; GDHO (−) N = 9). Importantly, disease-matched GDHO (+) and GDHO (–) EOC gene expression patterns remained distinct and provided improved group separation in this comparison (Figure 2c). At *p* < 0.01, 752 genes were differentially expressed, with 357 (47%) up-regulated in GDHO (+) EOC (Figure 2C; Table S1). Clustering of DEG using a false discovery rate (FDR) cutoff of 0.1 resulted in similar separation of disease-matched GDHO (+) vs. GDHO (−) EOC (Supplementary Figure S1b). A list of the DEG identified in each comparison is provided in Table S2. All genomic data from this study are deposited in the Gene Expression Omnibus (GEO) database, under accession number GSE146556.

**Figure 3:**
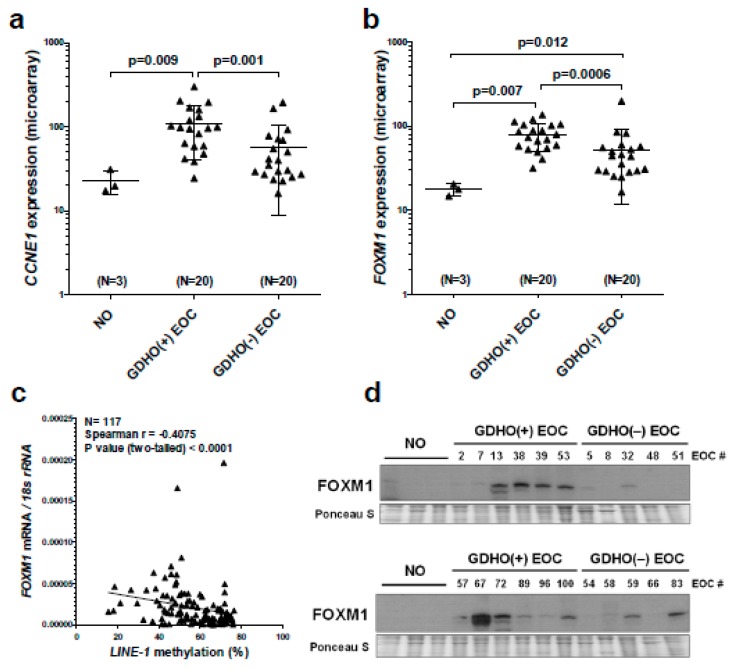
CCNE1 and FOXM1 are overexpressed in GDHO (+) EOC. (**a**) *CCNE1* expression in NO, GDHO (+) EOC, and GDHO (–) EOC, determined by Affymetrix microarray. (**b**) *FOXM1* expression in NO, GDHO (+) EOC, and GDHO (–) EOC, determined by Affymetrix microarray. Means ± SD are plotted, and Mann–Whitney p-values are indicated. (**c**) *FOXM1* mRNA expression vs. *LINE-1* methylation in an expanded set of EOC tumor samples. *FOXM1* expression was measured by RT-qPCR and *LINE-1* methylation was measured by pyrosequencing. Spearman test results and p-value are shown. (**d**) Western blot analysis of FOXM1 protein expression in NO, GDHO (+) EOC, and GDHO (–) EOC. The upper and lower blots are comprised of different sets of samples. Ponceau S staining is shown as a loading control.

We used Ingenuity Pathway Analyses (IPA) of expression data to interrogate cellular pathways altered in GDHO (+) tumors. We observed significant alterations in both cancer and reproductive disease pathways (Figure 2d; Table S3). In addition, functional pathways related to cell cycle, DNA replication, cell growth, and cell proliferation were remarkably altered. Thus, we suspected that increased proliferation was a key characteristic of GDHO (+) EOC (Figure 2d; Table S3). To test this, we measured the expression of the canonical proliferation marker *MKI67* [44] in GDHO (+) vs. GDHO (–) EOC. Both microarray and RT-qPCR analysis demonstrated significantly elevated *MKI67* expression in GDHO (+) EOC, as well as a significant negative association with *LINE-1* methylation (Supplementary Figure S2). In addition, two key oncogene drivers of EOC proliferation, *CCNE1* and *FOXM1* [38,45], were markedly up-regulated in GDHO (+) EOC (Figure 3a,b). An association of *CCNE1* amplification with DNA hypomethylation was previously reported in stomach cancer [46]. Based on the pervasive role of the FOXM1 pathway in EOC, and a report that FOXM1 is linked to changes in DNA methylation [38,47], we tested the association between *FOXM1* expression and *LINE-1* methylation in a larger set of EOC samples. Notably, we observed a strong inverse association between *FOXM1* expression and *LINE-1* methylation (Figure 3c). In addition to its mRNA, FOXM1 protein and several FOXM1 target genes, including *PLK1*, *AURKB*, *BIRC5*, and *CCNB1* [38], were significantly up-regulated in GDHO (+) EOC, consistent with functional activation of FOXM1 (Figure 3d; Figure S3). Furthermore, gene set enrichment analysis (GSEA) showed that there was significant enrichment of the FOXM1 transcription factor network (*p* < 0.01, FDR < 0.25) and G2/M checkpoint genes (*p* < 0.05, FDR < 0.25) in GDHO (+) EOC (data not shown). Together, these data implicate increased FOXM1 expression as a prominent feature of GDHO in EOC. We considered the possibility that increased expression of *CCNE1* and/or *FOXM1* in GDHO (+) EOC could result from direct hypomethylation of their promoters, i.e., correspond to a passenger effect in GDHO (+) EOC tumors. However, bisulfite sequencing analyses indicated that the *CCNE1* and *FOXM1* promoters were hypomethylated in NO, GDHO (–) and GDHO (+) EOC (Figure S4). Thus, CCNE1 and/or FOXM1 and, more generally, increased proliferation, might promote GDHO, rather than vice versa. 

**Figure 4:**
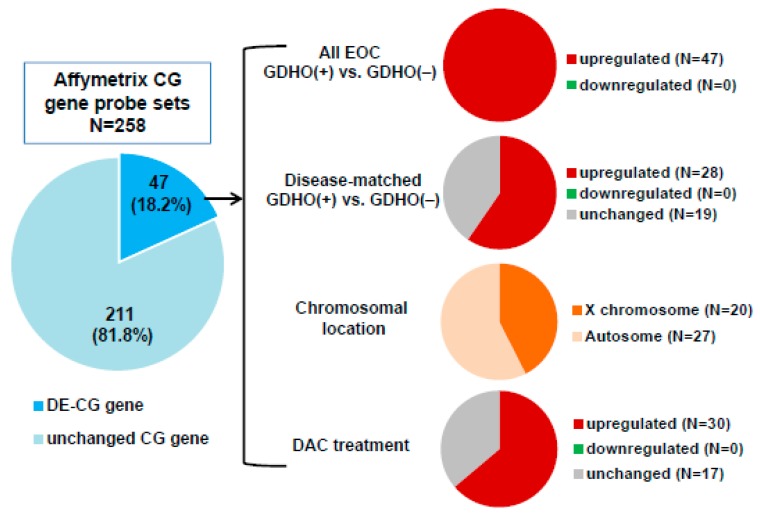
CG antigen genes are overexpressed in GDHO (+) EOC. A CG antigen gene list was obtained from the CT gene database [50]. Left: the proportion of CG genes (i.e., corresponding Affymetrix probes) differentially expressed between GDHO (+) and GDHO (–) EOC (*p* < 0.01) as determined by Affymetrix microarray. Right: Direction of expression change for the DEGs in all samples or disease-matched samples, their chromosomal localization, and the effect of decitabine (DAC) treatment on their expression in cell lines, as determined by Affymetrix microarray. See *Methods* for cell lines and treatment details.

GDHO (+) EOC tumors show altered expression of cancer-germline/cancer-testis (CG) genes, epigenetic regulators, and histone genes. Expression of CG genes in association with GDHO in cancer is well established, but most studies have investigated one or a few CG genes [8,9,10,12,13,40,48]. In contrast, the present data set allowed for a more comprehensive and global examination of this association. Roughly one-fifth of annotated CG genes were differentially expressed in GDHO (+) vs. GDHO (−) EOC, and each one was up-regulated in hypomethylated tumors (Figure 4; Table S4). In agreement, GSEA analysis of CG genes showed significant enrichment in GDHO (+) EOC (*p* < 0.01, FDR < 0.25). Over half of the CG genes activated in GDHO (+) EOC were induced in the disease-matched comparison, including both X-chromosome and autosomal CG genes (Figure 4; Table S4). The differentially expressed CG genes (DE-CG genes) included cancer vaccine targets and genes with oncogenic function, including *MAGEA*, *NY-ESO-1*, *XAGE-1*, *CT45,* and *PRAME* (Table S4). To test whether these genes are directly regulated by DNA methylation, we used Affymetrix microarrays to measure their expression in EOC cell models before and after decitabine (DAC) treatment [49]. Notably, the majority of the DE-CG genes were up-regulated by decitabine treatment (Figure 4; Table S4). These observations implicate DNA hypomethylation as a key driver of a sub-set of CG genes. 

Two additional gene groups of note showed altered expression in GDHO (+) EOC. First, genes with known roles in epigenetic regulation were altered. These genes included *EHMT2*/*G9a, ATAD2,* and *HDAC1*, which have reported oncogenic activity in EOC [51,52,53,54,55] (Table S5). Increased expression of *ATAD2* in GDHO (+) tumors could be due to promoter hypomethylation [56]. Interestingly, there was significantly increased expression of epigenetic regulators involved in gene activation (e.g., *JMJD2a*, *ATAD2*, *ASF1b*), and gene repression (e.g., *G9a*, *HDAC1*, *LSD1*) in GDHO (+) EOC (Table S5). In addition to epigenetic regulators, approximately half of all histone genes were up-regulated in GDHO (+) EOC vs. GDHO (−) EOC (Table S6). In part, this may reflect the increased proliferation signature observed in these tumors; in addition, direct hypomethylation of histone genes in EOC has been reported [57]. Bisulfite sequencing analysis of select histone genes indicated that some are constitutively hypomethylated in NO and EOC, while others are hypomethylated in GDHO (+) EOC vs. GDHO (−) EOC (Figure S5). 

### 2.3. DNA Methylome Characteristics of GDHO (+) EOC 

**Figure 5:**
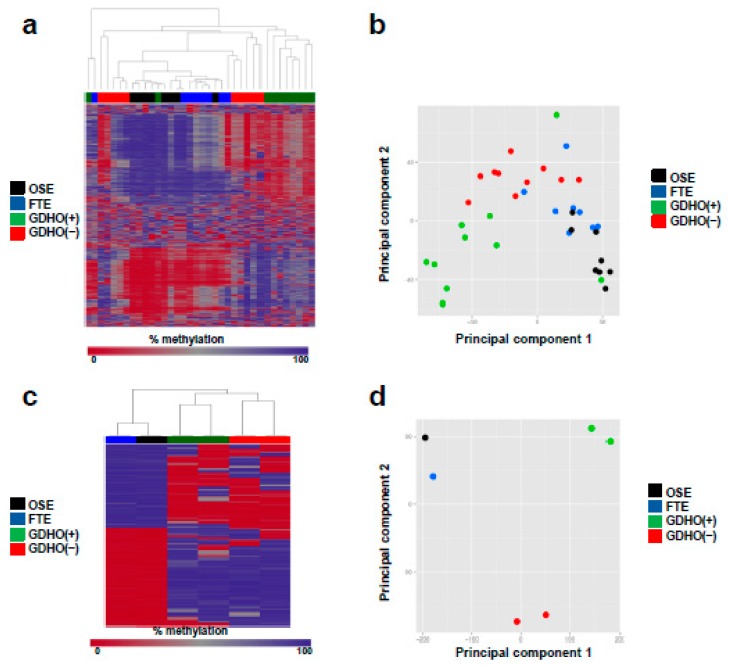

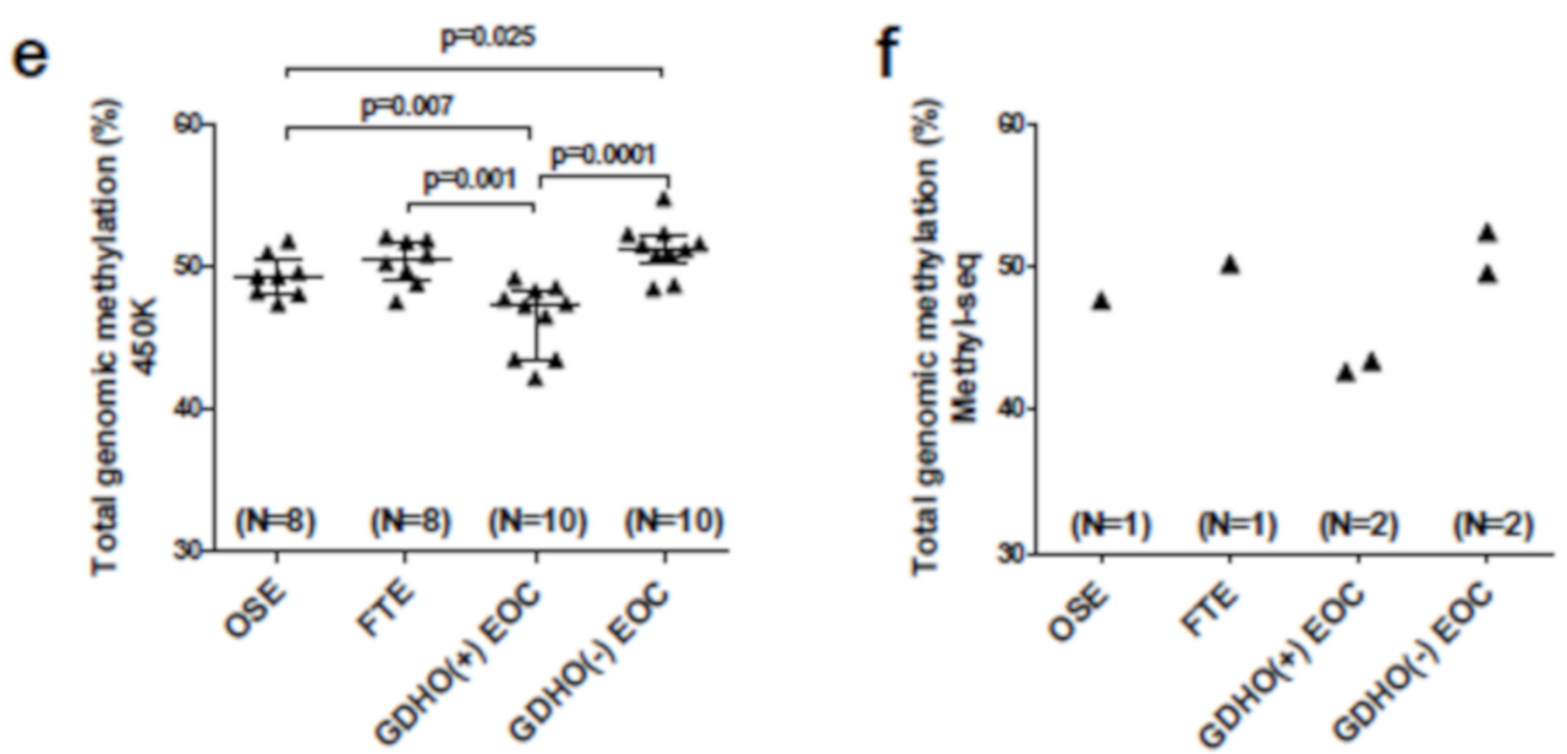
DNA methylome analysis of GDHO (+) EOC. (**a**) Dendogram showing relatedness of OSE, FTE, GDHO (+) EOC, and GDHO (–) EOC DNA methylomes, using Illumina 450K data. Data for the 1000 most variable CpG sites is shown. (**b**) Principal component analysis (PCA) of sample groups, using 450K data. (**c**) Dendogram showing relatedness of OSE, FTE, GDHO (+) EOC, and GDHO (–) EOC methylomes, using Methyl-seq data. The 1000 most variable CpG sites is shown. (**d**) PCA of sample groups using Methyl-seq data. (**e**) Total genomic methylation of OSE, FTE and EOC sample groups as determined using 450K data. Median and interquartile ranges are plotted, and the unpaired two-tailed t-test p-values are shown. (**f**) Total genomic methylation of OSE, FTE and EOC sample groups as determined using Methyl-seq data.

To better understand the DNA methylation landscape in GDHO (+) EOC, we initially used Illumina Infinium 450K arrays (450K) [58]. In addition to profiling both groups of EOC, we analyzed normal epithelia (NE; OSE + FTE average) as a control. Unsupervised hierarchical clustering and principal component analyses (PCA) of 450K data revealed that GDHO (+) and GDHO (–) EOC have distinct DNA methylomes, and, in addition that each are distinct from NE (Figure 5a,b). To define the methylome in greater depth, we next performed methylome sequencing (Methyl-seq) using a solution hybridization selection method [59]. 450K and Methyl-seq data were highly concordant (Pearson r = 0.97; N = 347,357 CpG sites), and hierarchical clustering and PCA analyses of Methyl-seq confirmed that GDHO (+) EOC, GDHO (–) EOC, and NE have distinct methylomes (Figure 5c,d). Compared to NE, DNA methylation was reduced in GDHO (+) but not GDHO (–) EOC (Figure 5e,f). 450K and Methyl-seq showed that a large number of promoters were differentially methylated in the two EOC groups, with most, regardless of CpG island context, showing hypomethylation in GDHO (+) EOC (Figure S6). In contrast, promoter hypermethylation and hypomethylation were similar in EOC vs. NE, with CpG island promoters mostly hypermethylated and non-CpG island promoters mostly hypomethylated in EOC (Figure S6). We defined differentially methylated regions (DMR) globally and found that the vast majority of DMR were hypomethylated in GDHO (+) vs. GDHO (−) EOC (Figure S7). The majority of DMR were also hypomethylated in EOC compared to NE (Figure S7). As methylation at different genomic locations has distinct consequences, we calculated differentially methylated CpG sites (DMC) independently for genes, CpG islands, CpG shores, CpG shelves, and CpG open seas, as described previously [58]. Most DMC were hypomethylated in GDHO (+) compared to GDHO (–) EOC, regardless of genomic context (Figure S7c,d), while hypermethylation and hypomethylation was more evenly split for EOC vs. NE (Figure S7e,f). In the latter comparison, CpG rich regions of the genome favored hypermethylation in EOC, while CpG poor regions (i.e., open seas) favored hypomethylation in EOC.

### 2.4. GDHO (+) EOC is Characterized by Hypomethylated Genomic Blocks 

**Figure 6:**
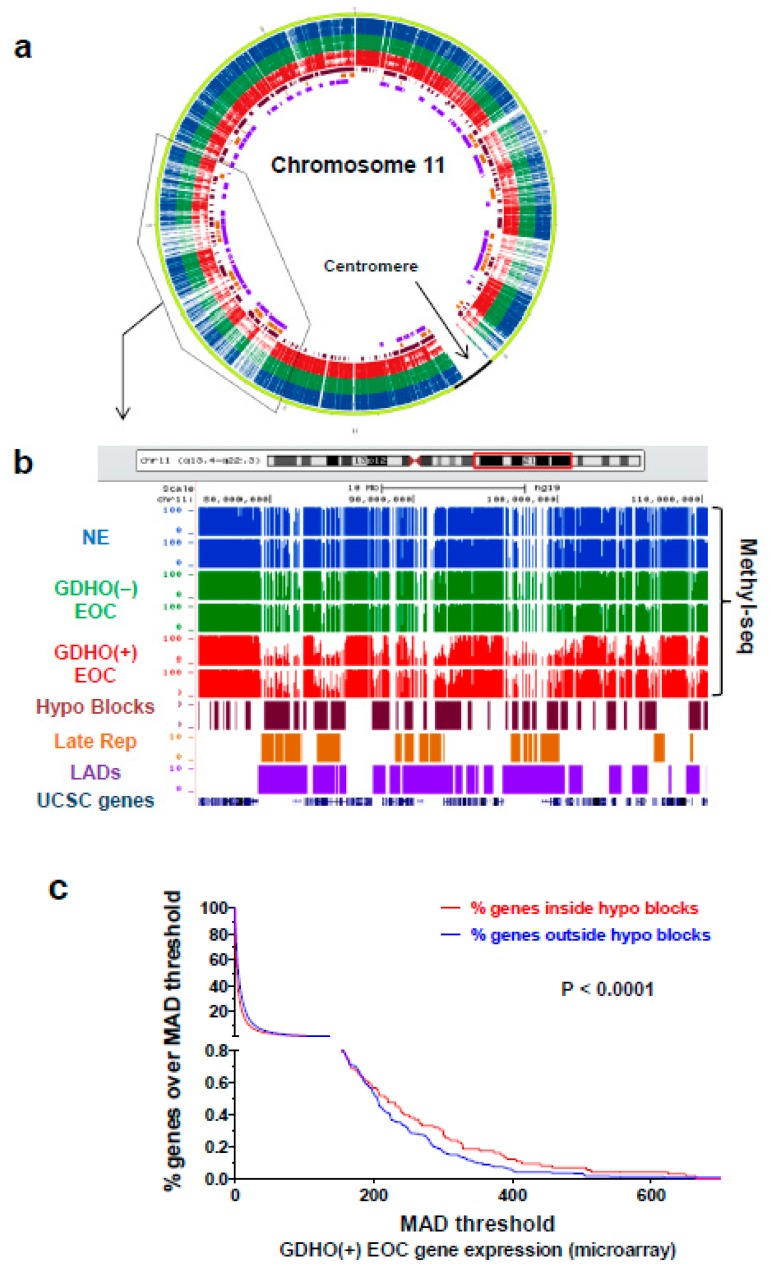
GDHO (+) EOC is characterized by hypomethylated genomic blocks. (**a**) Circos plot [60] of Methyl-seq data of chromosome 11 for OSE, FTE, GDHO (+) EOC, and GDHO (–) EOC. Methyl-seq data for each sample group, hypomethylated blocks, late-replicating regions, and LADs are indicated (see panel **b** for key). The enclosed region of the circos plot is enlarged in panel **b**. Chromosome 11 data is shown as an example. (**b**) Methyl-seq data for a selected region of chromosome 11 for OSE, FTE, GDHO (+) EOC, and GDHO (–) EOC. Calculated hypomethylated block regions are indicated at bottom, along with UCSC genome browser view of lamina-associated domains (LADs), late-replicating (Late rep) regions and gene positions. The Methyl-seq data is plotted on a scale from 0–100% methylation. (**c**) The relationship between hypomethylated blocks and gene expression variability in GDHO (+) EOC. Gene expression variability at hypomethylated blocks and non-block regions was determined using Affymetrix microarray data, by calculating the median average deviation (MAD) for individual gene expression values among the 20 GDHO (+) EOC samples. Hypomethylated blocks were calculated from Methyl-seq data by comparing GDHO (+) EOC to normal epithelia (NE; FTE + OSE average value). The 2-tailed paired t-test p-value is shown. The results indicate enrichment of hypervariable gene expression inside hypomethylated blocks.

To investigate whether hypomethylated blocks [14,15] are present in EOC, and to determine their relationship to GDHO, we first visually inspected Methyl-seq data using the *UCSC Genome Browser*. Chromosome 11 data are shown as a representative example (Figure 6a,b). These observational data revealed that hypomethylated blocks were both present and enriched in GDHO (+) EOC tumors. To formally test this over the entire genome, we used a quantitative approach to determine the number and size of hypomethylated blocks in the two EOC groups, using NE as a control (see *Methods*). While both 450K and Methyl-seq were capable of detecting and quantifying hypomethylated blocks (data not shown), we only present Methyl-seq data, due to its significantly greater genomic coverage. We observed large enrichment in both the number and size of hypomethylated blocks in GDHO (+) EOC vs. GDHO (–) EOC, with approximately 14% of the genome residing in hypomethylated blocks in the former (Table S7). Based on previous findings in other cancers, we quantified the overlap between EOC hypomethylated blocks, lamina-associated domains (LADs), and late-replicating genomic regions [16,28]. There was significant enrichment of hypomethylated blocks at LADs and late-replicating regions (Figure 6b; Table 1). Both genes and CpG islands were also enriched in hypomethylated blocks, but this might reflect the bias of our Methyl-seq method (Table 1; Table S8). We analyzed hypomethylated blocks for overlap with specific transcription factor (TF) binding sites, histone modifications, and RE, using ENCODE and the UCSC genome browser database. Among TF binding sites showing strong enrichment in EOC hypomethylated blocks were the polycomb repressor complex 2 (PRC2) components EZH2 and SUZ12, and the genomic insulator CTCF (Table 1; Table S9). Hypomethylated blocks were also highly enriched for H3K27me3, the modification recognized by PRC2 (Table 1; Table S10). These data suggest an association between DNA hypomethylation and repressive chromatin in EOC, as reported previously in breast cancer cells [17]. Notably, hypomethylated blocks were proposed to facilitate variable gene expression, providing a selective growth advantage during tumorigenesis [16]. In agreement, genes with high expression variability were enriched in the EOC hypomethylated blocks (Figure 6c). 

### 2.5. GDHO (+) EOC Tumors have Reduced Expression of Maintenance Methylation Components, when Normalized to Proliferation Markers 

**Figure 7:**
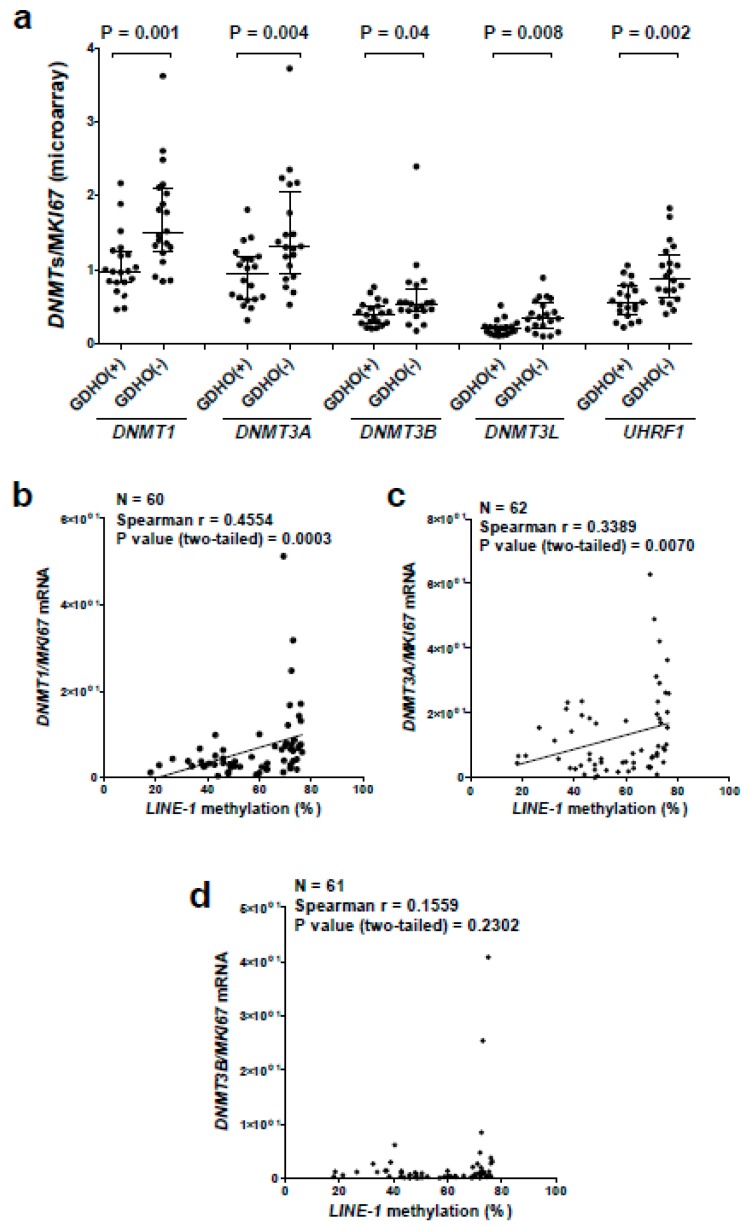
GDHO (+) EOC tumors show reduced expression of DNMTs and UHRF1 after normalization to proliferation. (**a**) Affymetrix gene expression data for *DNMT1*, *DNMT3A*, *DNMT3B*, *DNMT3L*, and *UHRF1*, normalized to *MKI67*, in GDHO (+) and GDHO (−) EOC. Median with interquartile range is plotted, and Mann–Whitney test p-values are shown. (**b**–**d**) Gene expression of (**b**) *DNMT1*, (**c**) *DNMT3A*, and (**d**) *DNMT3B*, normalized to *MKI67*, in EOC, as compared to *LINE-1* methylation in matched samples. Gene expression data were obtained by RT-qPCR, and *LINE-1* methylation was determined by bisulfite pyrosequencing. Spearman correlation analysis test results are shown.

The proliferative gene expression signature found in GDHO (+) EOC, coupled with the presence of hypomethylated blocks that overlap late-replicating regions, suggested a passive DNA hypomethylation mechanism. Paradoxically, however, elevated *DNMT* expression (relative to normal tissues) is commonly observed in cancer. To address this discrepancy, we first analyzed *DNMT* expression in our samples using standard Robust Multichip Average (RMA) Affymetrix normalization. This analysis method showed that *DNMT1* and *DNMT3B* were up-regulated in EOC compared to NO, while *DNMT3A* and *DNMT3L* were expressed at similar levels (Figure S8). However, we noted that maintenance DNA methylation is restricted to S phase, where *DNMT* expression is also increased [61]. Thus, we hypothesized that *DNMT* expression normalized to cell proliferation is a more relevant measure of maintenance methylation capacity in tumors. After normalization to the canonical cancer cell proliferation marker *MKI67* [44], all four DNMTs showed significantly reduced expression in EOC as compared to NO (Figure S9 and Figure S10), and in GDHO (+) vs. GDHO (–) EOC (Figure 7a). Moreover, *DNMT1* and *DNMT3A* expression, when normalized to *MKI67*, inversely correlated with *LINE-1* methylation in an expanded set of EOC samples (Figure 7b–d). In addition to DNMTs, UHRF1 is also a critical component of maintenance DNA methylation [62]. Similar to *DNMTs,* after normalization to *MKI67*, *UHRF1* was down-regulated in GDHO (+) compared to GDHO (−) EOC (Figure 7a). *UHRF1*, after *MKI67* normalization, also showed lower expression in EOC vs. NO (Figure S9e). Normalization of *DNMTs* and *UHRF1* to other markers of cancer cell proliferation, including *PLK1* and *BUB1* [44], provided similar results as seen with *MKI67* normalization (Figure S11 and Figure S12). These data suggest that GDHO (+) EOC tumors have reduced maintenance DNA methylation capacity.

### 2.6. GDHO (+) EOC Shows Increased Expression of Repetitive Elements (RE)

**Figure 8:**
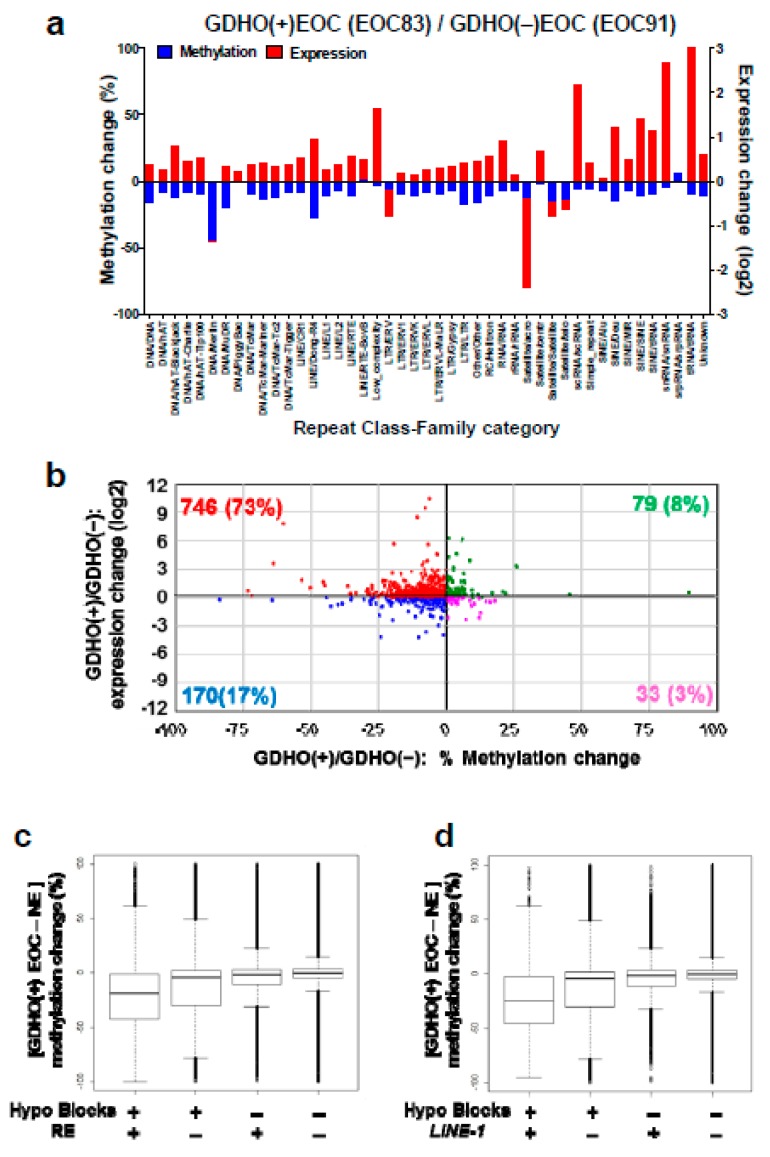
Overexpression of repetitive elements (RE) in GDHO (+) EOC. (**a**) Comparison of the expression and methylation of major sub-classes of REs in GDHO (+) vs. GDHO (–) EOC. Expression data were obtained from RNA-seq and methylation data from Methyl-seq. EOC83 and EOC91 are matched for stage and grade (stage 3C, grade 3). (**b**) RE expression vs. methylation in GDHO (+) vs. GDHO (–) EOC, using the same samples as in panel **a**. RE expression and methylation were determined by RNA-seq and Methyl-seq, respectively. Data points correspond to the average % methylation and log2 expression change across RE families. The results indicate that most RE families show increased expression and decreased methylation in GDHO (+) EOC. (**c**) DNA hypomethylation at hypomethylated blocks and/or all RE, in GDHO (+) as compared to NE, determined by Methyl-seq. (**d**) DNA hypomethylation at hypomethylated blocks and/or all *LINE-1* elements, in GDHO (+) as compared to NE, determined by Methyl-seq. In **c–d**, the median + interquartile range values are indicated. These data suggest that global hypomethylation is driven by hypomethylated block formation rather than *LINE-1* hypomethylation.

Based on prior knowledge [36], we investigated the relationship between GDHO and RE expression. We used total RNA-seq and Methyl-seq, methods that allow precise genomic mapping of repeat sequences. RE expression was mostly elevated in GDHO (+) EOC (Figure 8a,b). However, this effect was not uniform, but rather was class-specific (Figure 8a; Table S11). Most of the up-regulated RE in GDHO (+) EOC were hypomethylated at their corresponding genomic loci (Figure 8a,b). We additionally examined the relationship between RE methylation and hypomethylated blocks. RE showed specific patterns of enrichment or depletion in hypomethylated blocks, including *LINE-1*, which in most instances, was enriched in hypomethylated blocks. In contrast, *Alu/SINE* and satellite sequences were depleted from hypomethylated blocks (Table 1; Table S12). Methyl-seq data indicated that as reported previously for colon cancer [15], hypomethylation in EOC was mostly a consequence of hypomethylated block formation, rather than RE or *LINE-1* hypomethylation *per se* (Figure 8c,d). 

**Figure 9:**
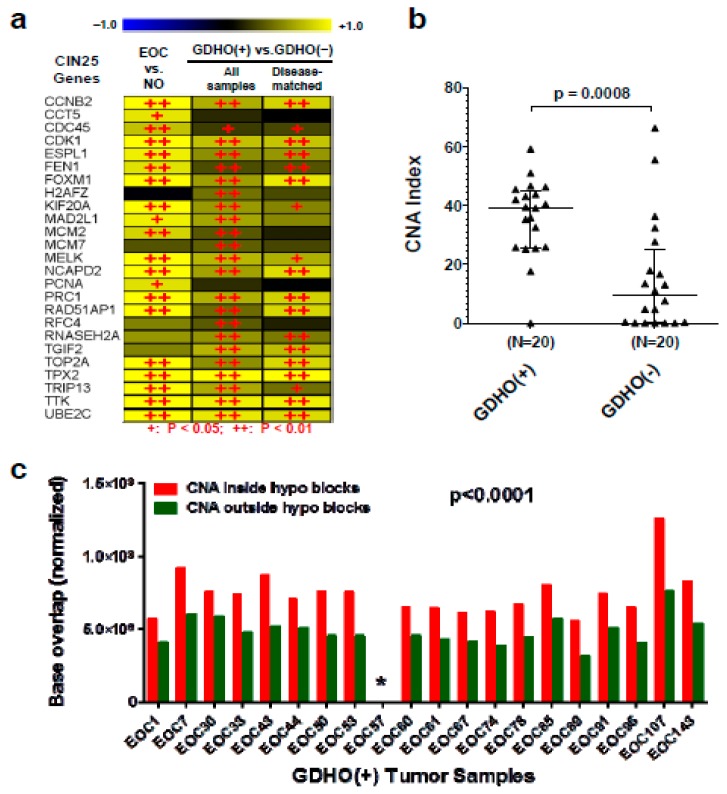
GDHO (+) EOC tumors show increased CIN, and CNA are enriched within hypomethylated blocks. (**a**) Expression of CIN25 signature genes in the indicated sample comparisons. Gene expression was determined by Affymetrix microarrays, and log2 fold change values are shown. + *p* < 0.05, ++ *p* < 0.01. (**b**) CNA index in GDHO (+) EOC vs. GDHO (–) EOC. EOC samples are disease-matched and represent HGSOC. CNA were determined using Affymetrix Cytoscan HD chips as described in *Methods*. Median with interquartile range is plotted, and the Mann–Whitney p-value is shown. (**c**) CNA are enriched in hypomethylated blocks. The total base pair overlap between CNA and hypomethylated blocks, or outside hypomethylated blocks, in GDHO (+) EOC. Hypomethylated blocks were determined by Methyl-seq analysis of GDHO (+) EOC vs. normal epithelia (NE; OSE + FTE average), and CNA were determined as described in panel **b**. Hypomethylated block and non-hypomethylated block regions were normalized according to the percent genomic coverage of each. The two-way ANOVA p-value is shown. The asterisk indicates that the values are too low to be visible on the scale.

GDHO (+) EOC tumors have increased chromosomal instability (CIN), and copy number alterations (CNA) are enriched in hypomethylated blocks. To further understand the consequences of GDHO, we focused on CIN, a hallmark of EOC [38]. We first used a previously reported 25-gene expression signature of CIN to interrogate the gene expression data [63]. The CIN25 signature was highly elevated in GDHO (+) vs. GDHO (–) EOC, both when analyzing all samples and in diseased-matched samples (Figure 9a). Next, we directly measured CNA in an additional set of 40 disease-matched GDHO (+) and GDHO (–) EOC (all HGSOC), using copy number/SNP arrays. This analysis revealed that CNA was significantly elevated in GDHO (+) EOC (Figure 9b). CNA was uniformly high in GDHO (+) EOC (with a single exception), while it was highly variable in GDHO (–) EOC (Figure 9b). These data suggest that other contributors to CIN are variably present in GDHO (−) EOC. Finally, we observed significant enrichment of CNA within the hypomethylated blocks (Figure 9c). This observation implicates hypomethylated blocks in the acquisition of CIN in EOC tumors.

## 3. Discussion

We report the evaluation of the molecular pathology associated with GDHO in EOC. We used *LINE-1* hypomethylation as a biomarker for GDHO based on earlier work [9], and observed that GDHO was associated with disease progression (advanced stage and grade) and reduced survival (disease-free and overall). By analyzing the mRNA transcriptome, RE expression, the DNA methylome, and copy number variation in parallel, we uncovered the major molecular features of GDHO in EOC. These included: (1) distinct patterns of gene expression, including activation of key drivers of EOC cell proliferation including *CCNE1* and *FOXM1*, (2) widespread activation of CG antigen genes, (3) deregulated expression of epigenetic regulators and histone genes, (4) increased expression of RE, often in conjunction with hypomethylation at the associated loci, and (5) increased CNA and CIN. Each of these features has oncogenic potential that are likely to contribute to disease progression and poor prognosis. In addition, DNA methylome analyses revealed the formation of hypomethylated blocks, which were not ubiquitous in EOC, but rather occurred most dramatically in GDHO (+) EOC. Importantly, all genes involved in maintenance DNA methylation displayed significantly reduced expression, relative to cell proliferation markers, in GDHO (+) EOC. Together, increased proliferation, hypomethylated block formation (which predominated at late-replicating regions), and decreased *DNMT* and *UHRF1* expression suggests that passive demethylation is a key contributor to GDHO in EOC. 

DNA hypomethylation was the first epigenetic alteration discovered in cancer [2]. Early studies showed that DNA hypomethylation can target oncogenes, coincides with overall loss of 5mdC, and is enriched in metastatic tumors [4,5,6,64]. Despite its early recognition, the mechanisms underlying GDHO are only recently emerging. The discovery of TET-assisted oxidative DNA demethylation, involving intermediates including 5-hydroxymethylcytosine (5hmC), suggests TET activation as a potential mechanism for GDHO. However, this appears unlikely, as TETs are often mutated or down-regulated in cancer, and cancer tissues show dramatically reduced levels of 5hmC [65]. Other possible mechanisms underlying GDHO have been proposed. For example, we reported that an increased (*BORIS*) *CTCFL/CTCF* ratio correlates with *LINE-1* hypomethylation in EOC [9]. However, BORIS overexpression was insufficient to promote DNA hypomethylation [66]. Other studies have suggested that UHRF1 overexpression (in hepatocellular carcinoma), and PIWI protein repression (in testicular cancer) contribute to GDHO [67,68]. However, in our data set, we found no evidence for these mechanisms. In contrast, we suggest that GDHO in EOC results, at least in part, from a proliferation-dependent process. The most statistically significant functional pathways up-regulated in GDHO involved cell proliferation, and, this was true for both unselected and disease-matched samples. We hypothesize that increased proliferation may overwhelm the capacity for EOC cells to perform efficient maintenance DNA methylation. Consistent with this model, a study of replication timing and DNA methylation found that late-replicating regions were hypomethylated and that this accumulated over cell divisions [69]. Also supportive of this model is the recent observation of a delay between DNA replication and the completion of maintenance methylation in cancer cells [70]. Finally, a recent landmark study demonstrated that DNA hypomethylation in cancer is concentrated at late-replicating domains partially methylated in normal tissues, and are enriched in solo “WCGW” CpG sites [28].

A passive model for GDHO predicts that DNA hypomethylation should be concentrated at the genomic regions that are the most difficult to methylate, e.g., heterochromatic and late replicating regions, including LADs; and this is precisely what we and others have observed [14,15,16,17,27,28]. The association of GDHO with LADs should be viewed with caution, however, as, to our knowledge, LADs have not been simultaneously mapped with DNA methylation in parallel. In this context, one report observed a link between DNA hypomethylation and late replication timing, and showed that the association of each of these sequences with LADs was secondary [71]. 

Because early data indicated that DNMT expression is often increased in cancer, it has long been assumed that DNMTs show a gain of function in cancer, which in turn might promote epigenetic silencing of tumor suppressor genes. However, this concept is paradoxical to the global reduction of methylation frequently observed in cancer [2]. Moreover, tumor suppressor functions for DNMTs have been demonstrated [72,73]. Because DNMTs are cell cycle regulated [61], and cancers, especially at later stages, often have an elevated proliferation index, we hypothesized that it is more appropriate to normalize DNMT expression to cell proliferation, in order to accurately gauge the functional capacity of maintenance methylation. Remarkably, after normalization to proliferation markers such as *MKI67*, we observed significantly reduced expression of all *DNMTs* and *UHFR1* in GDHO (+) EOC. In addition to *DNMT1*, reduced expression of *DNMT3A/3B/3L* would be expected to impair maintenance methylation, as these enzymes contribute to maintenance methylation of heterochromatic genomic regions [74,75]. In addition to reduced expression of DNMTs, a recent report noted that human cancers can show uncoordinated expression of these genes compared to normal tissues [76]. Although we did not measure DNMT protein expression, it was reported that DNMT protein expression is down-regulated in high malignant potential vs. low malignant potential EOC, and that reduced DNMT protein correlates with reduced EOC survival [77]. These observations are in agreement with our *DNMT* mRNA expression data, and are consistent with our observation that GDHO correlates with reduced EOC survival. 

The link observed between *FOXM1* and GDHO is of interest. While FOXM1′s association with GDHO may be indirect due to its role in promoting cell cycle progression, FOXM1 may be particularly relevant in HGSOC [38,45,78]. FOXM1 overexpression leads to DNA methylation changes in oral keratinocytes, including a global hypomethylation phenotype similar to that present in a squamous cell carcinoma cell line [47]. Coupled with our current data, an investigation of the potential mechanistic connection between FOXM1 and DNA hypomethylation in EOC appears warranted.

A seminal early study demonstrated that expression of the prototype CG antigen gene *MAGEA1* is linked to GDHO [8]. Cadieux et al. later reported a link between GDHO, *MAGEA1* expression, and cell proliferation in glioblastoma [79]. We measured expression of many CG genes in EOC using microarrays, and found that approximately one-fifth were activated in conjunction with GDHO. The lack of ubiquitous expression of all CG genes in GDHO (+) EOC suggests additional regulatory mechanisms, e.g., oncogenic transcription factors [10]. More generally, dramatically enhanced expression of CG antigens in association with GDHO suggests that patients harboring such tumors might be an optimal group for CG antigen-directed immunotherapy. Immune checkpoint modulators, such as α-CTLA4 or α-PD1/PD-L1 antibodies, require tumor antigens for their clinical activity [80]. Thus, these agents, as well as CG antigen vaccines, may be most effective in patients with GDHO (+) tumors [10,46,81,82]. It is relevant to test this concept in prospective clinical studies.

Pivotal work using murine models and human cell lines established a link between DNA hypomethylation and genomic instability [20,21]. This link is also observed in primary human tumors, but early studies focused on a limited number of CpG methylation sites and chromosomal alterations. More recently, large scale genomic approaches have been applied to this question and have reported clear associations of DNA methylation loss with various forms of cancer genomic instability [27,28,46]. In the current study, we comprehensively determined CNAs in HGSOC, which shows the most extreme level of CIN in human cancer [83]. GDHO (+) tumors had significantly higher levels of CIN vs. GDHO (−) tumors, and notably, CNAs were enriched in hypomethylated blocks. Consistently, chromosomal breakpoint regions in breast cancer cells co-localize with DMRs, which are typically hypomethylated in cancer [84]. Further understanding of the mechanistic relationship between DNA hypomethylation and genomic instability in cancer is warranted.

## 4. Methods

### 4.1. Human Tissues

NO, OSE, FTE, and EOC tissues were obtained from patients undergoing surgical resection at Roswell Park Comprehensive Cancer Center (RPCCC) under Institutional Review Board Protocol I-215512. These samples were described previously [9,43], and clinico-pathological information is provided in Table S13. NE controls (OSE and FTE) were obtained from patients without malignancy, and EOC samples contained > 90% neoplastic cells Processing of frozen tissue samples was described previously [40]. Table S14 lists the sample groups and the genomic analyses conducted on each.

### 4.2. DNA, RNA, and Protein Extractions 

Genomic DNA was isolated using the Puregene Tissue Kit (Qiagen Sciences, Inc., Germantown, MD, USA). Total RNA was purified using TRIzol (Invitrogen). Total cellular protein was extracted using RIPA buffer. Extractions were performed as described previously [9,40].

### 4.3. Cell lines and Drug Treatments 

IOSE-121 and OVCAR3 cells were described previously [48]. Cells at ~50% confluence were treated with 1 μM decitabine (DAC) (day 0), passaged at day 2, re-treated with 1 μM DAC at day 3, and harvested for RNA and DNA extractions at day 5. PBS was used as the vehicle control. The DAC treatment data in Figure 4 is compiled from treatment of these two cell lines.

### 4.4. Gene Expression Microarrays 

Affymetrix HG 1.0ST array analysis was performed at the University at Buffalo Center of Excellence in Bioinformatics and Life Sciences. Microarray probe cell intensity data (.cel) were normalized using the Affymetrix Expression Console (version 1.3.0.187, Thermo Fisher Scientific, Santa Clara, CA, USA) software running the RMA workflow. We used a regularized t-test analysis of control versus treatment comparisons using a Bayesian approach to estimate the within-treatment variation among replicates, using Cyber-T software. Expression heat maps were created using the TM4 microarray software suite Multi Experiment Viewer (MeV) software hierarchical clustering routine based on a Pearson correlation metric and average linkage [85]. Functional identification of gene networks was performed using Ingenuity Pathway Analysis (Qiagen Sciences Inc., Germantown, MD, USA). Gene set enrichment analysis (GSEA) [86] was used to determine the enrichment of the FOXM1 transcription factor network, G2/M checkpoint genes, and cancer-germline/cancer-testis (CG) genes [50].

### 4.5. Reverse Transcriptase-Quantitative PCR (RT-qPCR) 

RT-qPCR was performed as described previously [9]. Briefly, DNase-treated RNA was converted to cDNA, and samples were analyzed in triplicate using a BioRad CFX Connect system and the SYBR green method. Expression data were normalized to *18s rRNA*. Primer sequences are provided in Table S15. 

### 4.6. Western Blot Analysis 

Western blotting was performed as described previously [9]. We used the rabbit polyclonal primary anti-FOXM1 antibody (K19, Santa Cruz Biotechnology, sc-500, 1:500 dilution), and the goat anti-rabbit IgG-HRP secondary antibody (Santa Cruz Biotechnology, sc-2004, 1:5000 dilution). Ponceau S (Acros) staining was used as a loading control.

### 4.7. Bisulfite Clonal Sequencing and Pyrosequencing 

Genomic DNA was converted using the EZ DNA Methylation Kit (Zymo Research, Irvine, CA, USA). Bisulfite sequencing was accomplished as described previously [87] and DNA sequences were analyzed using Lasergene (DNASTAR). *LINE-1* pyrosequencing was performed as described previously [40], using the PSQ HS96 System ((Qiagen Sciences Inc., Germantown, MD, USA). Primer sequences are provided in Table S15. 

### 4.8. DNA Methylome Analyses 

Illumina Infinium 450K BeadChip analysis was performed at the RPCCC Genomics and the University of Utah Genomics Cores. Agilent SureSelect Methylome sequencing, a targeted solution hybridization bisulfite sequencing method (SHBS-seq) [59], was performed at the UNMC Epigenomics Core. This method encompasses ~15% of genomic CpG sites (3.5 × 10^6^ CpGs). High-throughput sequencing of library tags was performed at the UNMC Sequencing Core Facility, using an Illumina HiSeq 2000 Genome Analyzer. Sequence tags were aligned to the human genome (hg19) using the methylated sequence aligner Bismark [88]). The coverage of different genomic regions by Methyl-seq is shown in Table S8. Differentially methylated regions (DMR) corresponded to genomic regions of any length containing ≥ 3 CpG sites, with ≥ 1 CpG site showing a mean methylation change of >20% at *p* ≤ 0.05. RnBeads was used to analyze 450K and Methyl-seq data, to define methylation changes including DMR, promoters, CpG sites, and other genomic elements [89]. 

### 4.9. Determination of EOC Hypomethylated Blocks 

We defined hypomethylated blocks as described previously [16]. We used RnBeads to determine hypomethylated blocks using Methyl-seq data for NE control [(FTE + OSE average (N = 2)] versus GDHO (+) EOC (N = 2), using a ≤ 0.05 false discovery rate (FDR) of 5 kb tiled regions, with a ≥ 35% average methylation decrease. We combined regions that were ≤ 250kb distance apart, and selected final blocks containing ≥ 5 CpGs.

### 4.10. Correlation of EOC Hypomethylated Blocks with Genomic Features 

Spatial correlations were calculated between the reference hypomethylated block genomic intervals determined from GDHO (+) EOC Methyl-seq data and the query genomic intervals of specific genomic features, using the R package GenometriCorr [90]. CTCF, EZH2 and SUZ12 transcription binding sites were acquired from the UCSC Genome Browser hg19 table browser wgEncodeRegTfbsClusteredV3 table. *LINE-1*, *SINE-Alu,* and satellite repeat genomic locations were acquired from the UCSC Genome Browser hg19 table browser RepeatMasker. LAD genomic locations were acquired from the UCSC Genome Browser hg19 table browser NKI LADs (Tig3) database. Early and late-replicating genomic regions were acquired from the UCSC Genome Browser hg19 table browser Replication Timing by Repli-chip from ENCODE/FSU table IMR90 1, wgEncodeFsuRepliChipH1hescWaveSignalRep1. Histone modifications were acquired from the ENCODE ChIP-seq of mammary epithelial cells, using H3K9me3 (accession ENCFF001SWV), H3K4me3 (accession ENCFF001SXB), H3K27ac (accession ENCFF001SWW), H3K36me3 (accession ENCFF001SWY), and H3K27me3 (accession ENCFF001SWX).

### 4.11. Total RNA Sequencing (RNA-seq) 

RNA-seq was performed at the UNMC Sequencing Core Facility using the TruSeq Stranded Total RNA kit (Illumina) and an Illumina HiSeq 2000 Genome Analyzer. The starting material was 1.0 µg total RNA/sample. The resulting sequence tags were aligned to the UCSC Genome Browser reference human genome (hg19) mRNAs and REs using the software TopHat [91]. Cufflinks and Cuffdiff were used to estimate the expression values and determine differential expression of REs [91].

### 4.12. Genomic Copy Number Analysis 

Genomic copy number analysis was performed at the UNMC Sequencing Core Facility, using Affymetrix Cytoscan HD microarrays. The total number and size (segments) of CNA per sample were determined using the Affymetrix Chromosome Analysis Suite (Thermo Fisher Scientific, Santa Clara, CA, USA) Software. A CNA index was calculated for each sample based on the percent of the genome that resulted in either a copy number loss or gain. Base overlap (CNA inside hypomethylated blocks) and non-overlap (CNA outside hypomethylated blocks) between each EOC CNA segment and hypomethylated blocks was determined using the Bedtools intersect routine [92].

### 4.13. Correlation of LINE-1 Methylation with EOC Patient Survival 

Overall survival was defined as the number of months between the diagnosis date and death, and patients still alive were censored at their date of last follow-up. For disease-free survival, patients who were alive and disease-free were censored at the date of the last visit. *LINE-1* methylation, as determined by bisulfite pyrosequencing, was segregated into three groups as shown in Figure 1c,d, and survival was compared using Kaplan–Meier analyses. The null hypothesis of no difference in the survival distributions was assessed using the Logrank test. The age-independent association of *LINE-1* methylation with survival was tested using a proportional hazards model.

### 4.14. Genomic Data Deposit and Public Access

All genomic data from this project is available from the NCBI Gene Expression Omnibus (GEO) under accession number GSE146556.

## 5. Conclusions

In summary, this study provides new insight into the nature and consequences of global DNA hypomethylation in EOC. Future work should use this knowledge to assess the role of GDHO in the pathogenesis of EOC in vivo. In addition, future studies should define specific therapeutic approaches can be used to target tumors with GDHO.

## Figures and Tables

**Table 1:** Correlation of EOC hypomethylated blocks with genomic features ^1, 2.^

**Genomic Region**	**Number of Regions**	**Size of Regions (bp)**	**% of Genome**	**% Regions in Hypo Blocks**	**Correlation**	**Jaccard Test *P*-Value ^3,4^**
EOC Hypomethylated Blocks	2208	8.81E + 08	29	100	N/A	N/A
LADs	1231	1.13E + 09	37	36	Direct	<0.01
Early Replicating	910	7.10E + 08	23	25	Indirect	<0.01
Late-Replicating	1155	5.81E + 08	19	36	Direct	<0.01
CpG islands	27,537	2.10E + 07	1	32	Direct	<0.01
Genes	28,489	1.41E + 09	46	32	Direct	<0.01
	**# binding sites**	**# binding sites in hypo blocks**	**% binding sites in hypo blocks**	**Fold Enrichment**	**Correlation**	**Projection Test *P*-value**
EZH2 Sites	14,818	6103	41	1.41	Direct	<0.01
SUZ12 Sites	5772	2497	43	1.48	Direct	<0.01
CTCF Sites	162,209	52,216	32	1.10	Direct	<0.01
	**# peaks**	**# peaks in hypo blocks**	**% peaks in hypo blocks**	**Fold Enrichment**	**Correlation**	**Jaccard Test *P*-value**
H3K4me3	33,116	8655	26	0.90	Indirect	<0.01
H3K9me3	49,328	18,264	37	1.28	Direct	<0.01
H3K27ac	67,989	17,935	26	0.91	Indirect	<0.01
H3K27me3	40,126	15,428	38	1.32	Direct	<0.01
H3K36me3	33,342	6303	19	0.65	Indirect	<0.01
	**# of repeats**	**# repeats in hypo blocks**	**% repeats in hypo blocks**	**Fold Change**	**Correlation**	**Jaccard Test *P*-value**
*LINE-1*	951,780	264,352	28	0.96	Direct	<0.01
*Alu/SINE*	1,194,734	292,495	24	0.84	Indirect	<0.01
*Satellites*	6775	1052	16	0.64	Indirect	<0.01

1 Hypomethylated blocks were determined from Methyl-seq comparison of NE (OSE + FTE average) to GDHO(+) EOC; 2 Genomic features were retrieved from public databases, as described in *Methods;* 3 Statistical tests were performed using GenometriCorr (Favorov et al., *PLoS Computational Biology*, 2012), with hypomethylated block coordinates used as the reference value; 4 All tests, other than CpG islands, gave the same result when using hypomethylated blocks as query and the genomic features as the reference value. In contrast, the association with CpG islands was not significant.

## Supplementary Materials

The following are available online at https://www.mdpi.com/2072-6694/12/3/764/s1, Figure S1: Affymetrix gene expression of GDHO (+) vs. GDHO (-) EOC, Figure S2: *MKI67* gene expression, Figure S3: FOXM1 target gene expression in GDHO (+) vs. GDHO (-) EOC, Figure S4: Promoter bisulfite sequencing of *CCNE1* and *FOXM1*, Figure S5: Promoter bisulfite sequencing of histone genes, Figure S6: Differentially methylated promoters in GDHO (+) EOC, GDHO (-) EOC, and NE, Figure S7: Differentially methylated regions (DMR) and CpG sites (DMC) in GDHO (+) EOC, GDHO (-) EOC, and NE, Figure S8: *DNMT* and *UHRF1* gene expression in NO vs. EOC, Figure S9: *DNMT* and *UHRF1* Affymetrix gene expression normalized to *MKI67* in NO vs. EOC, Figure S10: RT-qPCR analysis of *DNMT* expression normalized to *MKI67* in NO vs. EOC, Figure S11: *DNMT* and *UHRF1* expression normalized to *PLK1* in NO, GDHO (+), and GDHO (-) EOC, Figure S12: *DNMT* and *UHRF1* expression normalized to *BUB1* in NO, GDHO (+), and GDHO (-) EOC, Table S1: Differentially expressed genes (DEG) in NO, EOC, GDHO(+), and GDHO(−) EOC, Table S2: Differentially expressed genes of all GDHO(+) EOC vs. GDHO(‒) EOC (P≤0.01), Table S3: Differentially expressed pathway of all DEGs (Ingenuity Pathway Analysis), Table S4: Differentially expressed CG antigen genes, Table S5: Differentially expressed epigenetic related genes of all GDHO(+) EOC vs. GDHO(‒) EOC (P≤0.05), Table S6: Differentially expressed histone genes of all GDHO(+) EOC vs. GDHO(‒) EOC (P≤0.05), Table S7: Hypomethylated blocks in GDHO(+) EOC vs. GDHO(−) EOC, Table S8: Agilent SureSelect Methyl-Seq Genomic Coverage, Table S9: Transcription factor binding sites in EOC hypomethylated blocks, Table S10: Histone marks in hypomethylated blocks, Table S11: RE expression and DNA methylation in EOC, Table S12: Repeat elements vs. hypo blocks in EOC, Table S13: EOC sample clinico-pathological information, Table S14: Sample groups and genomic/epigenomic analyses performed, Table S15: Primer sequences.

## Abbreviations

CG gene	cancer-germline or cancer-testis gene
CIN	chromosomal instability
CAN	copy number alteration
DAC	decitabine, 5-aza-2′-deoxycytidine
DEG	differentially expressed gene
DE-CG gene	differentially expressed CG gene
DMC	differentially methylated CpG site
DMR	differentially methylated region
DNMT	cytosine DNA methyltransferase
EOC	epithelial ovarian cancer
FDR	false discovery rate
FTE	fallopian tube epithelia
GDHO	global DNA hypomethylation
GSEA	gene set enrichment analysis
HGSOC	high-grade serous ovarian cancer
IPA	Ingenuity Pathway Analysis
LAD	lamina-associated domain
NE	normal epithelia (OSE + FTE)
NO	bulk normal ovary
OSE	ovarian surface epithelia
PCA	principal component analysis
RE	repetitive DNA elements
RMA	robust multichip average
TET	Ten-eleven translocation methylcytosine dioxygenase
TF	transcription factor

## References

[1] Jones P.A., Baylin S.B. (2007). The epigenomics of cancer. Cell.

[2] Feinberg A.P., Tycko B. (2004). The history of cancer epigenetics. Nat. Rev. Cancer.

[3] Widschwendter M., Fiegl H., Egle D., Mueller-Holzner E., Spizzo G., Marth C., Weisenberger D.J., Campan M., Young J., Jacobs I. (2007). Epigenetic stem cell signature in cancer. Nat. Genet..

[4] Feinberg A.P., Gehrke C.W., Kuo K.C., Ehrlich M. (1988). Reduced genomic 5-methylcytosine content in human colonic neoplasia. Cancer Res..

[5] Bedford M.T., van Helden P.D. (1987). Hypomethylation of DNA in pathological conditions of the human prostate. Cancer Res..

[6] Gama-Sosa M.A., Slagel V.A., Trewyn R.W., Oxenhandler R., Kuo K.C., Gehrke C.W., Ehrlich M. (1983). The 5-methylcytosine content of DNA from human tumors. Nucleic Acids Res..

[7] Beck C.R., Garcia-Perez J.L., Badge R.M., Moran J.V. (2011). LINE-1 elements in structural variation and disease. Annu. Rev. Genom. Hum. Genet..

[8] De Smet C., De Backer O., Faraoni I., Lurquin C., Brasseur F., Boon T. (1996). The activation of human gene MAGE-1 in tumor cells is correlated with genome-wide demethylation. Proc. Natl. Acad. Sci. USA.

[9] Woloszynska-Read A., Zhang W., Yu J., Link P.A., Mhawech-Fauceglia P., Collamat G., Akers S.N., Ostler K.R., Godley L.A., Odunsi K. (2011). Coordinated cancer germline antigen promoter and global DNA hypomethylation in ovarian cancer: Association with the BORIS/CTCF expression ratio and advanced stage. Clin. Cancer Res..

[10] Akers S.N., Odunsi K., Karpf A.R. (2010). Regulation of cancer germline antigen gene expression: Implications for cancer immunotherapy. Future Oncol..

[11] Sharma A., Albahrani M., Zhang W., Kufel C.N., James S.R., Odunsi K., Klinkebiel D., Karpf A.R. (2019). Epigenetic activation of POTE genes in ovarian cancer. Epigenetics.

[12] Zhang W., Barger C.J., Eng K.H., Klinkebiel D., Link P.A., Omilian A., Bshara W., Odunsi K., Karpf A.R. (2016). PRAME expression and promoter hypomethylation in epithelial ovarian cancer. Oncotarget.

[13] Zhang W., Barger C.J., Link P.A., Mhawech-Fauceglia P., Miller A., Akers S.N., Odunsi K., Karpf A.R. (2015). DNA hypomethylation-mediated activation of Cancer/Testis Antigen 45 (CT45) genes is associated with disease progression and reduced survival in epithelial ovarian cancer. Epigenetics.

[14] Berman B.P., Weisenberger D.J., Aman J.F., Hinoue T., Ramjan Z., Liu Y., Noushmehr H., Lange C.P., van Dijk C.M., Tollenaar R.A. (2012). Regions of focal DNA hypermethylation and long-range hypomethylation in colorectal cancer coincide with nuclear lamina-associated domains. Nat. Genet..

[15] Hansen K.D., Timp W., Bravo H.C., Sabunciyan S., Langmead B., McDonald O.G., Wen B., Wu H., Liu Y., Diep D. (2011). Increased methylation variation in epigenetic domains across cancer types. Nat. Genet..

[16] Timp W., Bravo H.C., McDonald O.G., Goggins M., Umbricht C., Zeiger M., Feinberg A.P., Irizarry R.A. (2014). Large hypomethylated blocks as a universal defining epigenetic alteration in human solid tumors. Genome Med..

[17] Hon G.C., Hawkins R.D., Caballero O.L., Lo C., Lister R., Pelizzola M., Valsesia A., Ye Z., Kuan S., Edsall L.E. (2012). Global DNA hypomethylation coupled to repressive chromatin domain formation and gene silencing in breast cancer. Genome Res..

[18] Hinoue T., Weisenberger D.J., Lange C.P., Shen H., Byun H.M., Van Den Berg D., Malik S., Pan F., Noushmehr H., van Dijk C.M. (2012). Genome-scale analysis of aberrant DNA methylation in colorectal cancer. Genome Res..

[19] Schulz W.A., Steinhoff C., Florl A.R. (2006). Methylation of endogenous human retroelements in health and disease. Curr. Top. Microbiol. Immunol..

[20] Karpf A.R., Matsui S. (2005). Genetic disruption of cytosine DNA methyltransferase enzymes induces chromosomal instability in human cancer cells. Cancer Res..

[21] Eden A., Gaudet F., Waghmare A., Jaenisch R. (2003). Chromosomal instability and tumors promoted by DNA hypomethylation. Science.

[22] Gaudet F., Hodgson J.G., Eden A., Jackson-Grusby L., Dausman J., Gray J.W., Leonhardt H., Jaenisch R. (2003). Induction of tumors in mice by genomic hypomethylation. Science.

[23] Rodriguez J., Frigola J., Vendrell E., Risques R.A., Fraga M.F., Morales C., Moreno V., Esteller M., Capella G., Ribas M. (2006). Chromosomal instability correlates with genome-wide DNA demethylation in human primary colorectal cancers. Cancer Res..

[24] Suzuki K., Suzuki I., Leodolter A., Alonso S., Horiuchi S., Yamashita K., Perucho M. (2006). Global DNA demethylation in gastrointestinal cancer is age dependent and precedes genomic damage. Cancer Cell.

[25] Daskalos A., Nikolaidis G., Xinarianos G., Savvari P., Cassidy A., Zakopoulou R., Kotsinas A., Gorgoulis V., Field J.K., Liloglou T. (2009). Hypomethylation of retrotransposable elements correlates with genomic instability in non-small cell lung cancer. Int. J. Cancer..

[26] Richards K.L., Zhang B., Baggerly K.A., Colella S., Lang J.C., Schuller D.E., Krahe R. (2009). Genome-wide hypomethylation in head and neck cancer is more pronounced in HPV-negative tumors and is associated with genomic instability. PLOS ONE.

[27] Du Q., Bert S.A., Armstrong N.J., Caldon C.E., Song J.Z., Nair S.S., Gould C.M., Luu P.L., Peters T., Khoury A. (2019). Replication timing and epigenome remodelling are associated with the nature of chromosomal rearrangements in cancer. Nat. Commun..

[28] Zhou W., Dinh H.Q., Ramjan Z., Weisenberger D.J., Nicolet C.M., Shen H., Laird P.W., Berman B.P. (2018). DNA methylation loss in late-replicating domains is linked to mitotic cell division. Nat. Genet..

[29] Epping M.T., Wang L., Edel M.J., Carlee L., Hernandez M., Bernards R. (2005). The human tumor antigen PRAME is a dominant repressor of retinoic acid receptor signaling. Cell.

[30] Karpf A.R., Bai S., James S.R., Mohler J.L., Wilson E.M. (2009). Increased expression of androgen receptor coregulator MAGE-11 in prostate cancer by DNA hypomethylation and cyclic AMP. Mol. Cancer Res..

[31] Oricchio E., Sciamanna I., Beraldi R., Tolstonog G.V., Schumann G.G., Spadafora C. (2007). Distinct roles for LINE-1 and HERV-K retroelements in cell proliferation, differentiation and tumor progression. Oncogene.

[32] Hillman J.C., Pugacheva E.M., Barger C.J., Sribenja S., Rosario S., Albahrani M., Truskinovsky A.M., Stablewski A., Liu S., Loukinov D.I. (2019). BORIS Expression in Ovarian Cancer Precursor Cells Alters the CTCF Cistrome and Enhances Invasiveness through GALNT14. Mol. Cancer Res..

[33] Timp W., Feinberg A.P. (2013). Cancer as a dysregulated epigenome allowing cellular growth advantage at the expense of the host. Nat. Rev. Cancer.

[34] Wild L., Flanagan J.M. (2010). Genome-wide hypomethylation in cancer may be a passive consequence of transformation. Biochim. Biophys. Acta.

[35] Eswaran J., Horvath A., Godbole S., Reddy S.D., Mudvari P., Ohshiro K., Cyanam D., Nair S., Fuqua S.A., Polyak K. (2013). RNA sequencing of cancer reveals novel splicing alterations. Sci. Rep..

[36] Ting D.T., Lipson D., Paul S., Brannigan B.W., Akhavanfard S., Coffman E.J., Contino G., Deshpande V., Iafrate A.J., Letovsky S. (2011). Aberrant overexpression of satellite repeats in pancreatic and other epithelial cancers. Science.

[37] Vaughan S., Coward J.I., Bast R.C., Berchuck A., Berek J.S., Brenton J.D., Coukos G., Crum C.C., Drapkin R., Etemadmoghadam D. (2011). Rethinking ovarian cancer: Recommendations for improving outcomes. Nat. Rev. Cancer.

[38] (2011). Integrated genomic analyses of ovarian carcinoma. Nature.

[39] Nephew K.P., Balch C., Zhang S., Huang T.H. (2009). Epigenetics and ovarian cancer. Cancer Treat. Res..

[40] Woloszynska-Read A., Mhawech-Fauceglia P., Yu J., Odunsi K., Karpf A.R. (2008). Intertumor and intratumor NY-ESO-1 expression heterogeneity is associated with promoter-specific and global DNA methylation status in ovarian cancer. Clin. Cancer Res..

[41] Widschwendter M., Jiang G., Woods C., Muller H.M., Fiegl H., Goebel G., Marth C., Muller-Holzner E., Zeimet A.G., Laird P.W. (2004). DNA hypomethylation and ovarian cancer biology. Cancer Res..

[42] Pisanic T.R., Asaka S., Lin S.F., Yen T.T., Sun H., Bahadirli-Talbott A., Wang T.H., Burns K.H., Wang T.L., Shih I.M. (2019). Long Interspersed Nuclear Element 1 Retrotransposons Become Deregulated during the Development of Ovarian Cancer Precursor Lesions. Am. J. Pathol..

[43] Akers S.N., Moysich K., Zhang W., Collamat Lai G., Miller A., Lele S., Odunsi K., Karpf A.R. (2014). LINE1 and Alu repetitive element DNA methylation in tumors and white blood cells from epithelial ovarian cancer patients. Gynecol. Oncol..

[44] Whitfield M.L., George L.K., Grant G.D., Perou C.M. (2006). Common markers of proliferation. Nat. Rev. Cancer.

[45] Barger C.J., Zhang W., Hillman J., Stablewski A.B., Higgins M.J., Vanderhyden B.C., Odunsi K., Karpf A.R. (2015). Genetic determinants of FOXM1 overexpression in epithelial ovarian cancer and functional contribution to cell cycle progression. Oncotarget.

[46] Saghafinia S., Mina M., Riggi N., Hanahan D., Ciriello G. (2018). Pan-Cancer Landscape of Aberrant DNA Methylation across Human Tumors. Cell Rep..

[47] Teh M.T., Gemenetzidis E., Patel D., Tariq R., Nadir A., Bahta A.W., Waseem A., Hutchison I.L. (2012). FOXM1 induces a global methylation signature that mimics the cancer epigenome in head and neck squamous cell carcinoma. PLOS ONE.

[48] Woloszynska-Read A., James S.R., Link P.A., Yu J., Odunsi K., Karpf A.R. (2007). DNA methylation-dependent regulation of BORIS/CTCFL expression in ovarian cancer. Cancer Immun..

[49] Karpf A.R., Jones D.A. (2002). Reactivating the expression of methylation silenced genes in human cancer. Oncogene.

[50] Almeida L.G., Sakabe N.J., de Oliveira A.R., Silva M.C., Mundstein A.S., Cohen T., Chen Y.T., Chua R., Gurung S., Gnjatic S. (2009). CTdatabase: a knowledge-base of high-throughput and curated data on cancer-testis antigens. Nucleic Acids Research.

[51] Hua K.T., Wang M.Y., Chen M.W., Wei L.H., Chen C.K., Ko C.H., Jeng Y.M., Sung P.L., Jan Y.H., Hsiao M. (2014). The H3K9 methyltransferase G9a is a marker of aggressive ovarian cancer that promotes peritoneal metastasis. Mol. Cancer.

[52] Wan W.N., Zhang Y.X., Wang X.M., Liu Y.J., Zhang Y.Q., Que Y.H., Zhao W.J. (2014). ATAD2 is highly expressed in ovarian carcinomas and indicates poor prognosis. Asian Pac. J. Cancer Prev..

[53] Hayashi A., Horiuchi A., Kikuchi N., Hayashi T., Fuseya C., Suzuki A., Konishi I., Shiozawa T. (2010). Type-specific roles of histone deacetylase (HDAC) overexpression in ovarian carcinoma: HDAC1 enhances cell proliferation and HDAC3 stimulates cell migration with down-regulation of E-cadherin. Int. J. Cancer..

[54] Yang G., Chang B., Yang F., Guo X., Cai K.Q., Xiao X.S., Wang H., Sen S., Hung M.C., Mills G.B. (2010). Aurora kinase A promotes ovarian tumorigenesis through dysregulation of the cell cycle and suppression of BRCA2. Clin. Cancer Res..

[55] Jimeno A., Li J., Messersmith W.A., Laheru D., Rudek M.A., Maniar M., Hidalgo M., Baker S.D., Donehower R.C. (2008). Phase I study of ON 01910.Na, a novel modulator of the Polo-like kinase 1 pathway, in adult patients with solid tumors. J. Clin. Oncol..

[56] Wrzeszczynski K.O., Varadan V., Byrnes J., Lum E., Kamalakaran S., Levine D.A., Dimitrova N., Zhang M.Q., Lucito R. (2011). Identification of tumor suppressors and oncogenes from genomic and epigenetic features in ovarian cancer. PLOS ONE.

[57] Liao Y.P., Chen L.Y., Huang R.L., Su P.H., Chan M.W., Chang C.C., Yu M.H., Wang P.H., Yen M.S., Nephew K.P. (2014). Hypomethylation signature of tumor-initiating cells predicts poor prognosis of ovarian cancer patients. Hum. Mol. Genet..

[58] Sandoval J., Heyn H., Moran S., Serra-Musach J., Pujana M.A., Bibikova M., Esteller M. (2011). Validation of a DNA methylation microarray for 450,000 CpG sites in the human genome. Epigenetics.

[59] Lee E.J., Pei L., Srivastava G., Joshi T., Kushwaha G., Choi J.H., Robertson K.D., Wang X., Colbourne J.K., Zhang L. (2011). Targeted bisulfite sequencing by solution hybrid selection and massively parallel sequencing. Nucleic Acids Res..

[60] Krzywinski M., Schein J., Birol I., Connors J., Gascoyne R., Horsman D., Jones S.J., Marra M.A. (2009). Circos: an information aesthetic for comparative genomics. Genome Research.

[61] Robertson K.D., Keyomarsi K., Gonzales F.A., Velicescu M., Jones P.A. (2000). Differential mRNA expression of the human DNA methyltransferases (DNMTs) 1, 3a and 3b during the G(0)/G(1) to S phase transition in normal and tumor cells. Nucleic Acids Res..

[62] Bostick M., Kim J.K., Esteve P.O., Clark A., Pradhan S., Jacobsen S.E. (2007). UHRF1 plays a role in maintaining DNA methylation in mammalian cells. Science.

[63] Carter S.L., Eklund A.C., Kohane I.S., Harris L.N., Szallasi Z. (2006). A signature of chromosomal instability inferred from gene expression profiles predicts clinical outcome in multiple human cancers. Nat. Genet..

[64] Feinberg A.P., Vogelstein B. (1983). Hypomethylation distinguishes genes of some human cancers from their normal counterparts. Nature.

[65] Pfeifer G.P., Xiong W., Hahn M.A., Jin S.G. (2014). The role of 5-hydroxymethylcytosine in human cancer. Cell Tissue Res..

[66] Woloszynska-Read A., James S.R., Song C., Jin B., Odunsi K., Karpf A.R. (2010). BORIS/CTCFL expression is insufficient for cancer-germline antigen gene expression and DNA hypomethylation in ovarian cell lines. Cancer Immun..

[67] Ferreira H.J., Heyn H., Garcia Del Muro X., Vidal A., Larriba S., Munoz C., Villanueva A., Esteller M. (2013). Epigenetic loss of the PIWI/piRNA machinery in human testicular tumorigenesis. Epigenetics.

[68] Mudbhary R., Hoshida Y., Chernyavskaya Y., Jacob V., Villanueva A., Fiel M.I., Chen X., Kojima K., Thung S., Bronson R.T. (2014). UHRF1 Overexpression Drives DNA Hypomethylation and Hepatocellular Carcinoma. Cancer Cell.

[69] Aran D., Toperoff G., Rosenberg M., Hellman A. (2011). Replication timing-related and gene body-specific methylation of active human genes. Hum. Mol. Genet..

[70] Desjobert C., El Mai M., Gerard-Hirne T., Guianvarc’h D., Carrier A., Pottier C., Arimondo P.B., Riond J. (2014). Combined analysis of DNA methylation and cell cycle in cancer cells. Epigenetics.

[71] Shipony Z., Mukamel Z., Cohen N.M., Landan G., Chomsky E., Zeliger S.R., Fried Y.C., Ainbinder E., Friedman N., Tanay A. (2014). Dynamic and static maintenance of epigenetic memory in pluripotent and somatic cells. Nature.

[72] Peters S.L., Hlady R.A., Opavska J., Klinkebiel D., Pirruccello S.J., Talmon G.A., Sharp J.G., Wu L., Jaenisch R., Simpson M.A. (2014). Tumor suppressor functions of Dnmt3a and Dnmt3b in the prevention of malignant mouse lymphopoiesis. Leukemia.

[73] Kinney S.R., Moser M.T., Pascual M., Greally J.M., Foster B.A., Karpf A.R. (2010). Opposing roles of Dnmt1 in early and late-stage murine prostate cancer. Mol. Cell. Biol..

[74] Jeong S., Liang G., Sharma S., Lin J.C., Choi S.H., Han H., Yoo C.B., Egger G., Yang A.S., Jones P.A. (2009). Selective anchoring of DNA methyltransferases 3A and 3B to nucleosomes containing methylated DNA. Mol. Cell. Biol..

[75] Wen B., Wu H., Shinkai Y., Irizarry R.A., Feinberg A.P. (2009). Large histone H3 lysine 9 dimethylated chromatin blocks distinguish differentiated from embryonic stem cells. Nat. Genet..

[76] Liu J., Cui X., Jiang J., Cao D., He Y., Wang H. (2017). Uncoordinated expression of DNA methylation-related enzymes in human cancer. Epigenetics Chromatin.

[77] Bai X., Song Z., Fu Y., Yu Z., Zhao L., Zhao H., Yao W., Huang D., Mi X., Wang E. (2012). Clinicopathological significance and prognostic value of DNA methyltransferase 1, 3a, and 3b expressions in sporadic epithelial ovarian cancer. PLOS ONE.

[78] Barger C.J., Branick C., Chee L., Karpf A.R. (2019). Pan-Cancer Analyses Reveal Genomic Features of FOXM1 Overexpression in Cancer. Cancers.

[79] Cadieux B., Ching T.T., VandenBerg S.R., Costello J.F. (2006). Genome-wide hypomethylation in human glioblastomas associated with specific copy number alteration, methylenetetrahydrofolate reductase allele status, and increased proliferation. Cancer Res..

[80] Coulie P.G., Van den Eynde B.J., van der Bruggen P., Boon T. (2014). Tumour antigens recognized by T lymphocytes: At the core of cancer immunotherapy. Nat. Rev. Cancer.

[81] Odunsi K., Matsuzaki J., James S.R., Mhawech-Fauceglia P., Tsuji T., Miller A., Zhang W., Akers S.N., Griffiths E.A., Miliotto A. (2014). Epigenetic potentiation of NY-ESO-1 vaccine therapy in human ovarian cancer. Cancer Immunol. Res..

[82] Karpf A.R. (2006). A potential role for epigenetic modulatory drugs in the enhancement of cancer/germ-line antigen vaccine efficacy. Epigenetics.

[83] Ciriello G., Miller M.L., Aksoy B.A., Senbabaoglu Y., Schultz N., Sander C. (2013). Emerging landscape of oncogenic signatures across human cancers. Nat. Genet..

[84] Tang M.H., Varadan V., Kamalakaran S., Zhang M.Q., Dimitrova N., Hicks J. (2012). Major chromosomal breakpoint intervals in breast cancer co-localize with differentially methylated regions. Front. Oncol..

[85] Saeed A.I., Bhagabati N.K., Braisted J.C., Liang W., Sharov V., Howe E.A., Li J., Thiagarajan M., White J.A., Quackenbush J. (2006). TM4 microarray software suite. Methods Enzymol.

[86] Subramanian A., Tamayo P., Mootha V.K., Mukherjee S., Ebert B.L., Gillette M.A., Paulovich A., Pomeroy S.L., Golub T.R., Lander E.S. (2005). Gene set enrichment analysis: A knowledge-based approach for interpreting genome-wide expression profiles. Proc. Natl. Acad. Sci. USA.

[87] James S.R., Link P.A., Karpf A.R. (2006). Epigenetic regulation of X-linked cancer/germline antigen genes by DNMT1 and DNMT3b. Oncogene.

[88] Krueger F., Andrews S.R. (2011). Bismark: a flexible aligner and methylation caller for Bisulfite-Seq applications. Bioinformatics.

[89] Assenov Y., Muller F., Lutsik P., Walter J., Lengauer T., Bock C. (2014). Comprehensive analysis of DNA methylation data with RnBeads. Nat. Methods.

[90] Favorov A., Mularoni L., Cope L.M., Medvedeva Y., Mironov A.A., Makeev V.J., Wheelan S.J. (2012). Exploring massive, genome scale datasets with the GenometriCorr package. PLoS Comput. Biol..

[91] Trapnell C., Roberts A., Goff L., Pertea G., Kim D., Kelley D.R., Pimentel H., Salzberg S.L., Rinn J.L., Pachter L. (2012). Differential gene and transcript expression analysis of RNA-seq experiments with TopHat and Cufflinks. Nat. Protoc..

[92] Quinlan A.R. (2014). BEDTools: The Swiss-Army Tool for Genome Feature Analysis. Curr Protoc Bioinformatics.

