# Supplementary material for: Global DNA Hypomethylation in Epithelial Ovarian Cancer: Passive Demethylation and Association with Genomic Instability
Source: Cancers (Basel). 2020 Mar 24;12(3):764. doi: 10.3390/cancers12030764 (PMC7140107; doi:10.3390/cancers12030764)
Supplement: Supplementary file 1 [file cancers-12-00764-s001.zip › Supplemental Figures.pdf]

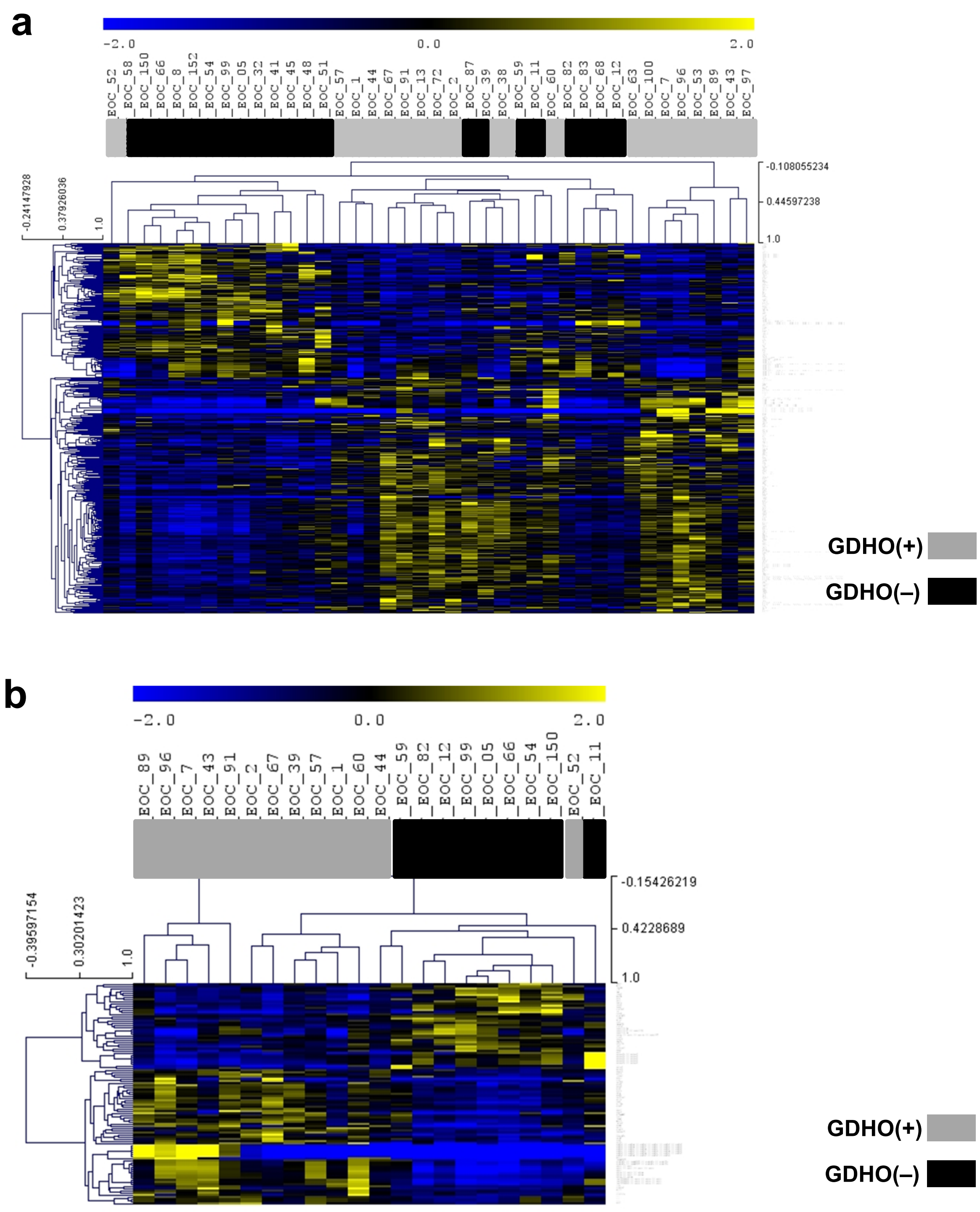


**Zhang et al. Supplementary Fig. 1. Affymetrix gene expression of GDHO(+) vs. GDHO(−) EOC. (a)** Hierarchical clustering heat map of DEGs (FDR≤0.1) between GDHO(+) vs. GDHO(–) EOC (all samples). **(b)** Hierarchical clustering heat map of DEGs (FDR≤0.1) between disease-matched GDHO(+) vs. GDHO(–) EOC.

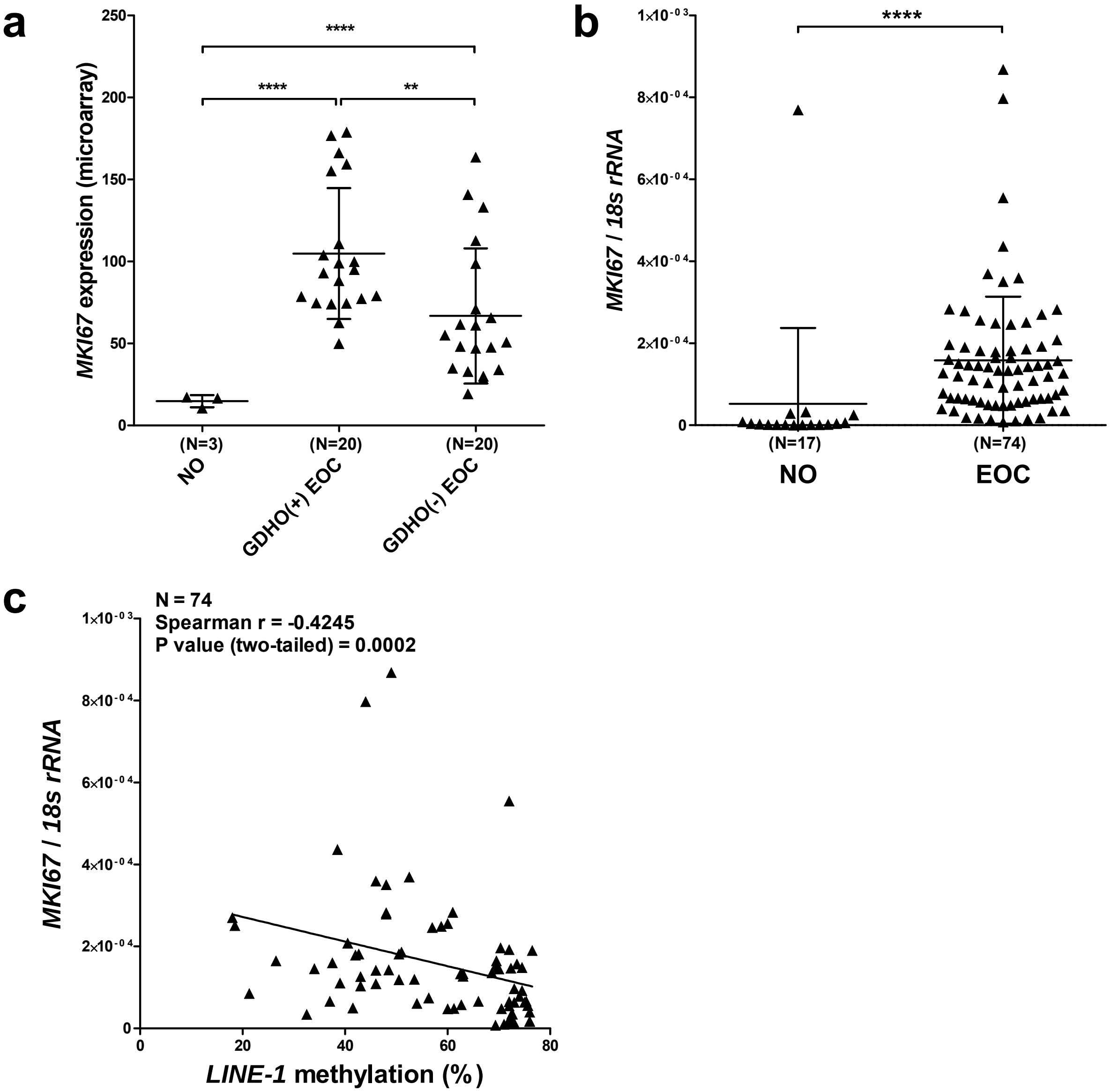


**Zhang et al. Supplementary Fig. 2.** **(a)** *MKI67* expression in NO, GDHO(+) EOC, and GDHO(−) EOC as determined by Affymetrix microarray. **(b)** *MKI67* expression in NO and EOC as determined by RT-qPCR. For **a** and **b**, mean +/− SD are plotted, and the two-tailed Mann-Whitney test p-values are indicated (**p<0.01, ****p<0.0001). **(c)** *MKI67* expression vs. *LINE-1* methylation in EOC. *MKI67* expression was determined by RT-qPCR and *LINE-1* methylation was determined by pyrosequencing. The Spearman correlation test results are indicated.

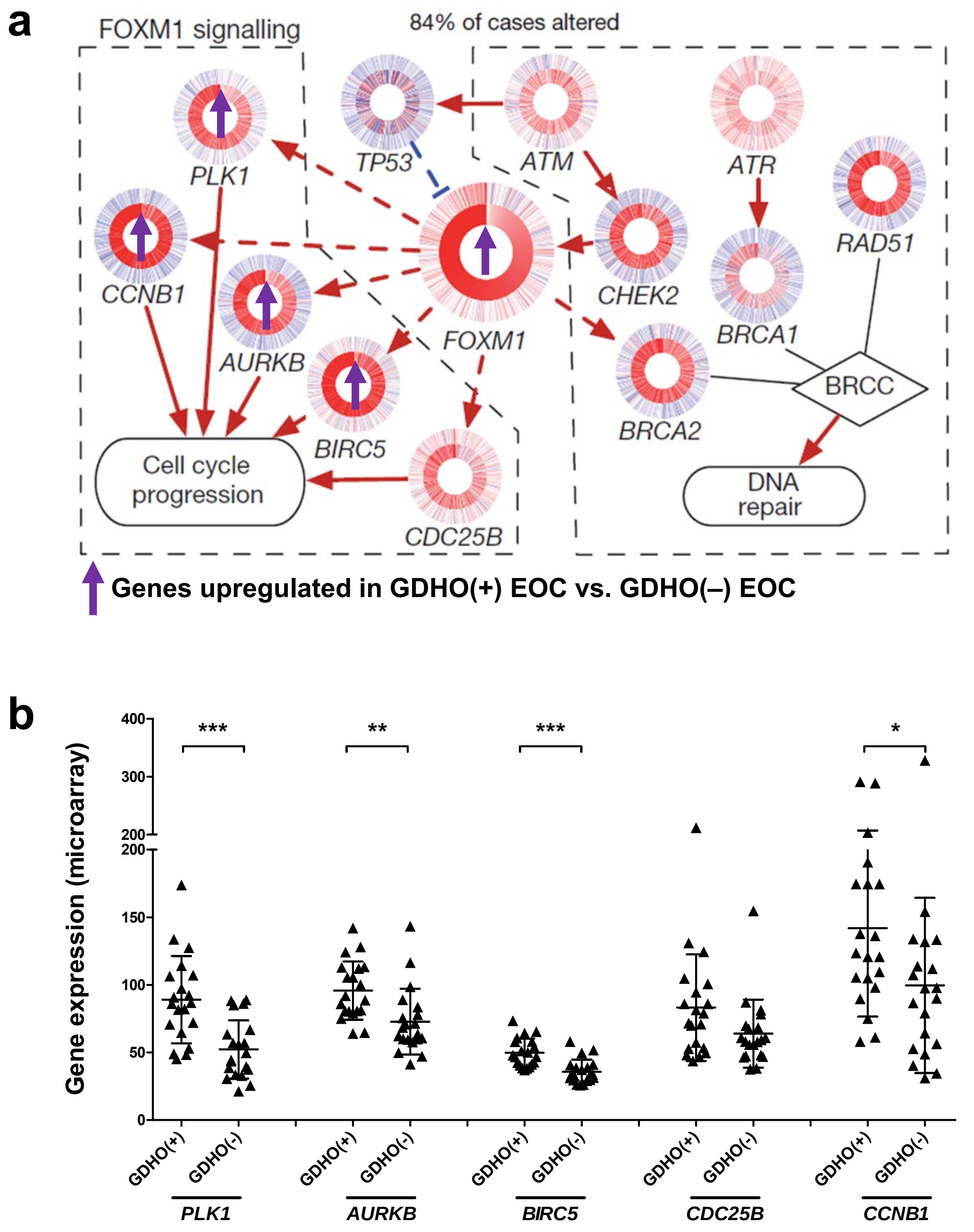


**Zhang et al. Supplementary Fig. 3. FOXM1 target gene expression in GDHO(+) EOC vs. GDHO(–) EOC. (a)** Schematic of *FOXM1* pathway activation in GDHO(+) EOC vs. GDHO(–) EOC, as determined by Affymetrix microarray. Purple upward arrows indicate significantly upregulated genes. Figure adapted from: *Integrated genomic analyses of ovarian carcinoma. Nature 474, 609-615, doi:10.1038/nature10166 (2011).* **(b)** *FOXM1* target gene expression data in GDHO(+) EOC and GDHO(–) EOC, as determined by Affymetrix microarray. Mean +/− SD are plotted, and two-tailed Mann-Whitney test p-values are indicated (*p<0.05, **p<0.01, ***p<0.001).

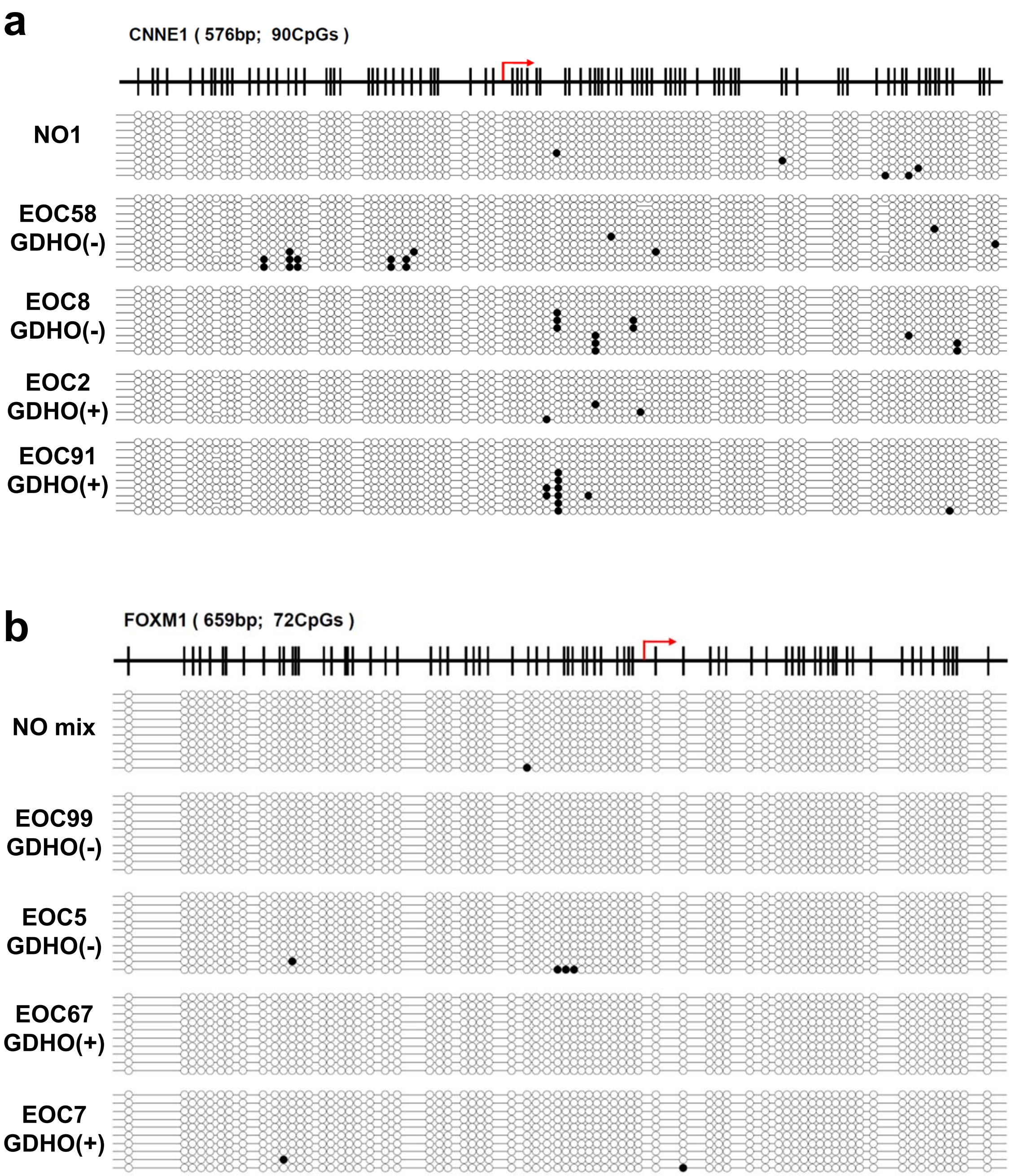


**Zhang et al. Supplementary Fig. 4. Promoter bisulfite sequencing of *CCNE1* and *FOXM1*.** Bisulfite clonal sequencing results of **(a)** *CCNE1* and **(b)** *FOXM1*. NCBI indicated transcription start sites (red broken arrows) and CpG sites (black hash marks) are indicated in the promoter region diagrams at top. Filled and open circles indicate methylated and unmethylated CpG sites, respectively, and each row represents one sequenced allele. Data are shown for NO (NO1 or admix of 3 NO), two GDHO(+) EOC, and two GDHO(–) EOC samples.

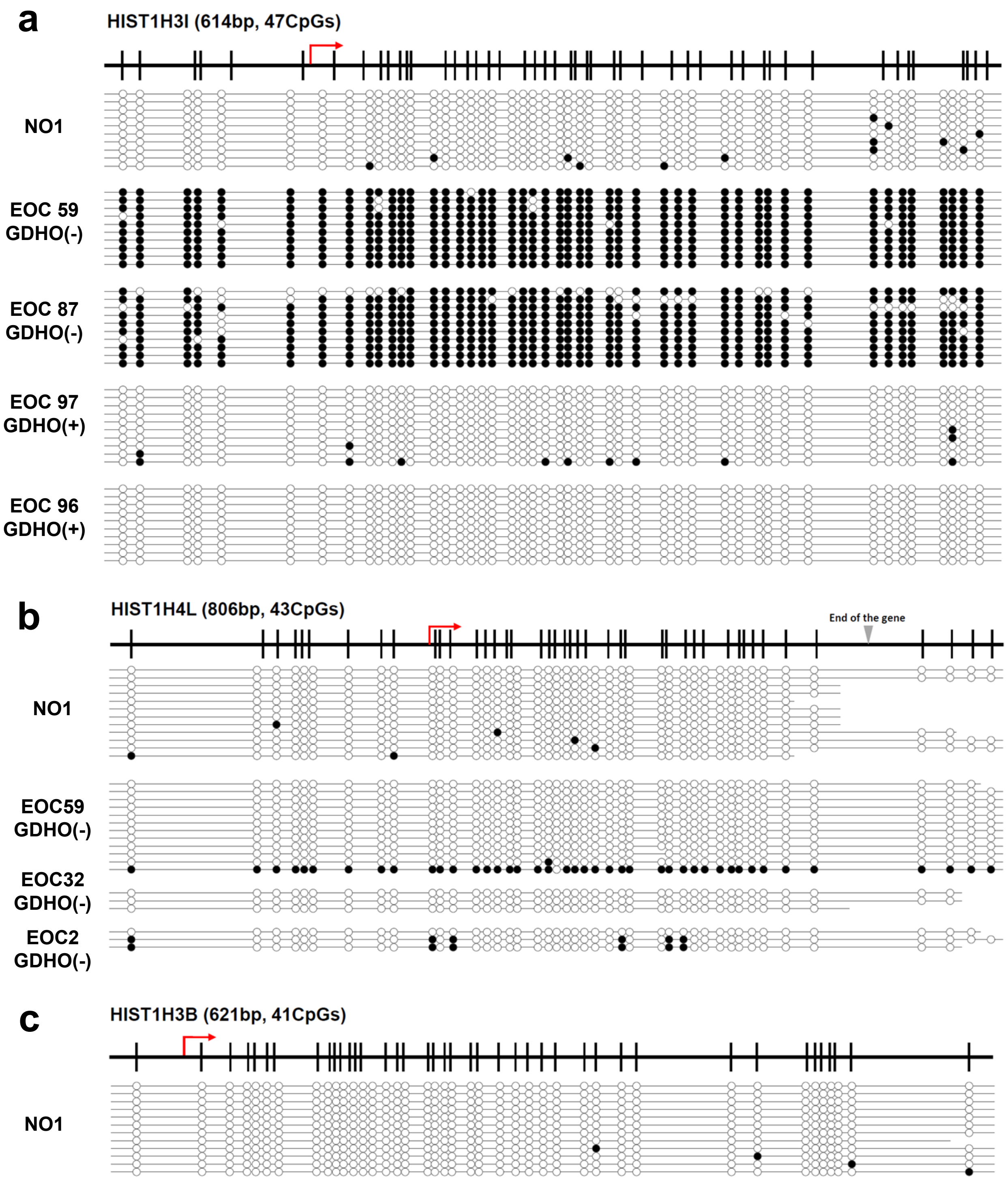


**Zhang et al. Supplementary Fig. 5. Promoter bisulfite sequencing of histone genes.** Bisulfite clonal sequencing results for **(a)** *HIST1H3I*, **(b)** *HIST1H4L*, and **(c)** *HIST1H3B*. NCBI indicated transcription start sites (red broken arrows) and CpG sites (black hash marks) are indicated in the promoter region diagrams at top. Filled and open circles indicate methylated and unmethylated CpG sites, respectively, and each row represents one sequenced allele. Data are shown for NO (NO1), GDHO(+) EOC and GDHO(–) EOC samples.

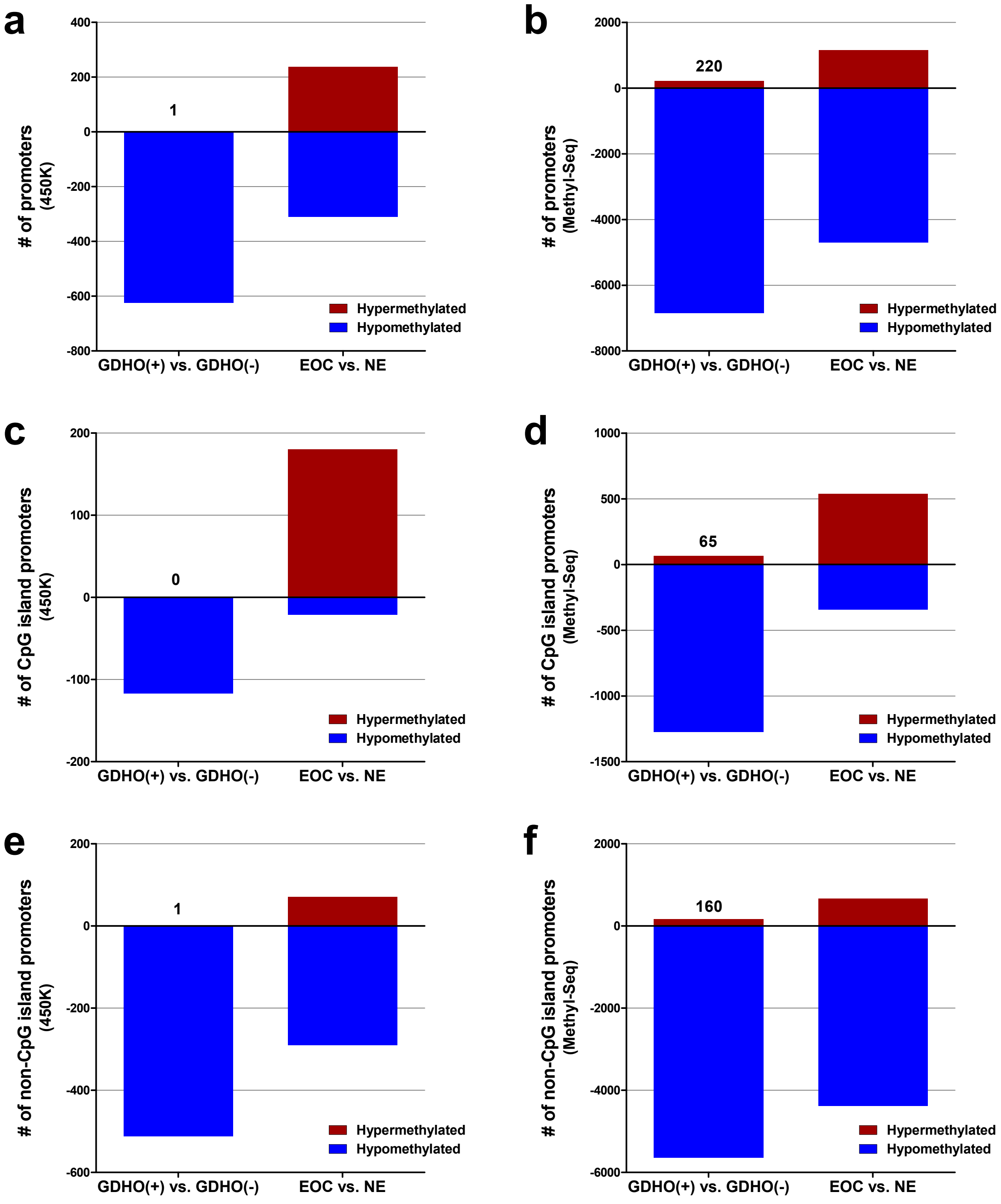


**Zhang et al. Supplementary Fig. 6. Differentially methylated promoters in GDHO(+) EOC, GDHO(−) EOC, and NE.** **(a)** Total number of promoters showing differential methylation in the indicated comparisons, and the direction of methylation change, as determined by 450K analysis. **(b)** Same as **a**, but data were obtained from Methyl-seq. **(c-d)** Same as a-b, but data are shown only for CpG island containing promoters. **(e-f)** Same as a-b, but data are shown only for non-CpG island promoters. Differentially methylated promoters were defined as promoters containing ≥ 3 CpGs, ≥ 20% methylation change, and p-value ≤ 0.05.

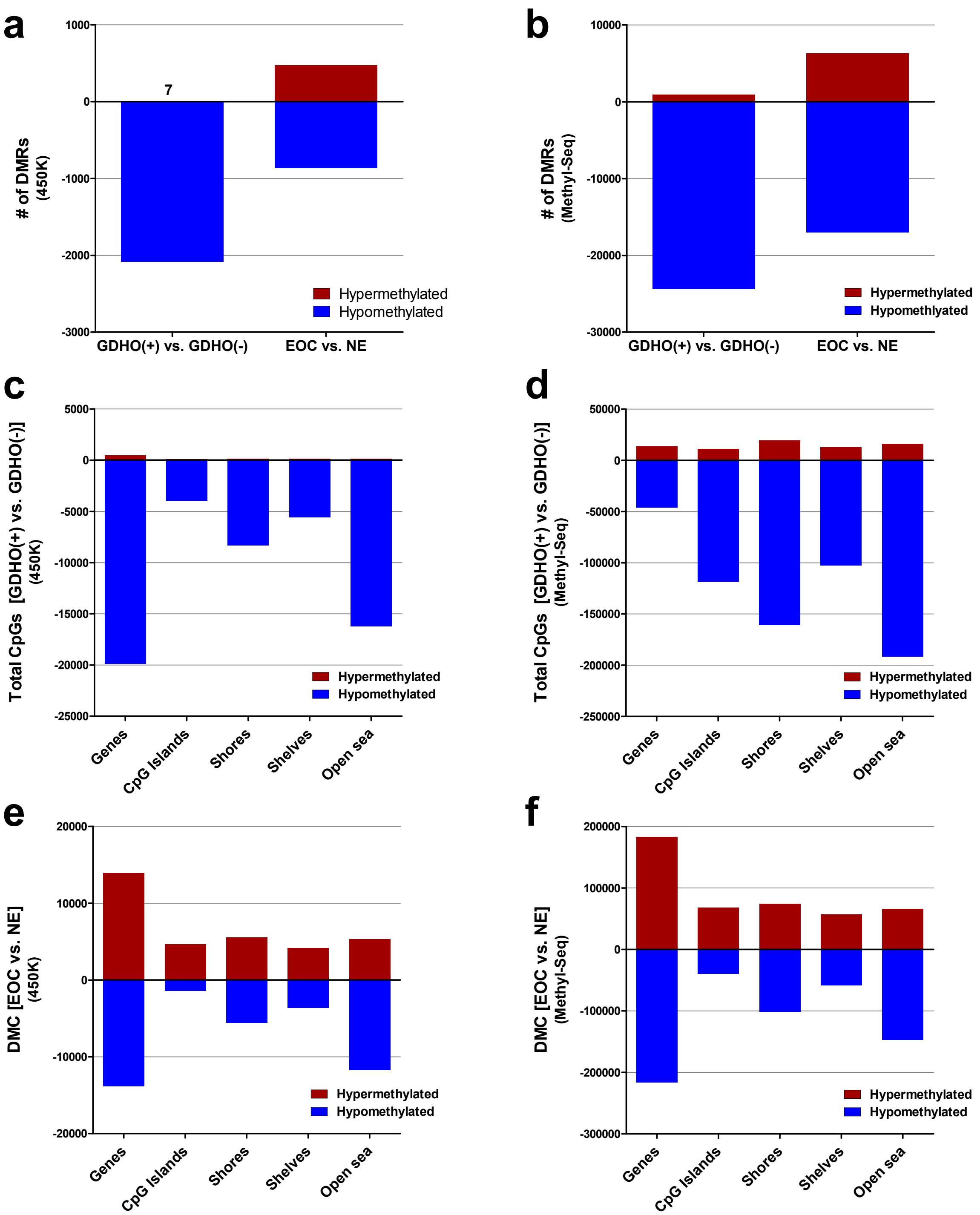


**Zhang et al. Supplementary Fig. 7. Differentially methylated regions (DMR) and CpG sites (DMC) in GDHO(+) EOC, GDHO(−) EOC, and NE. (a)** Total number of DMR in the indicated comparisons, and the direction of methylation change, as determined by 450K. **(b)** Same as **a** but using Methyl-seq data. DMR were defined as regions ≥ 3 CpGs ≥ 20% methylation change and p-value ≤ 0.05. **(c)** DMC between GDHO(+) and GDHO(−) EOC for the indicated genomic regions, as determined by 450K. **(d)** Same as **c**, but using Methyl-seq data. **(e)** DMC between EOC and NE for the indicated genomic regions, as determined by 450K. **(f)** Same as **e**, but using Methyl-seq data. DMC were defined as CpGs showing ≥ 20% methylation change and p-value ≤ 0.05.

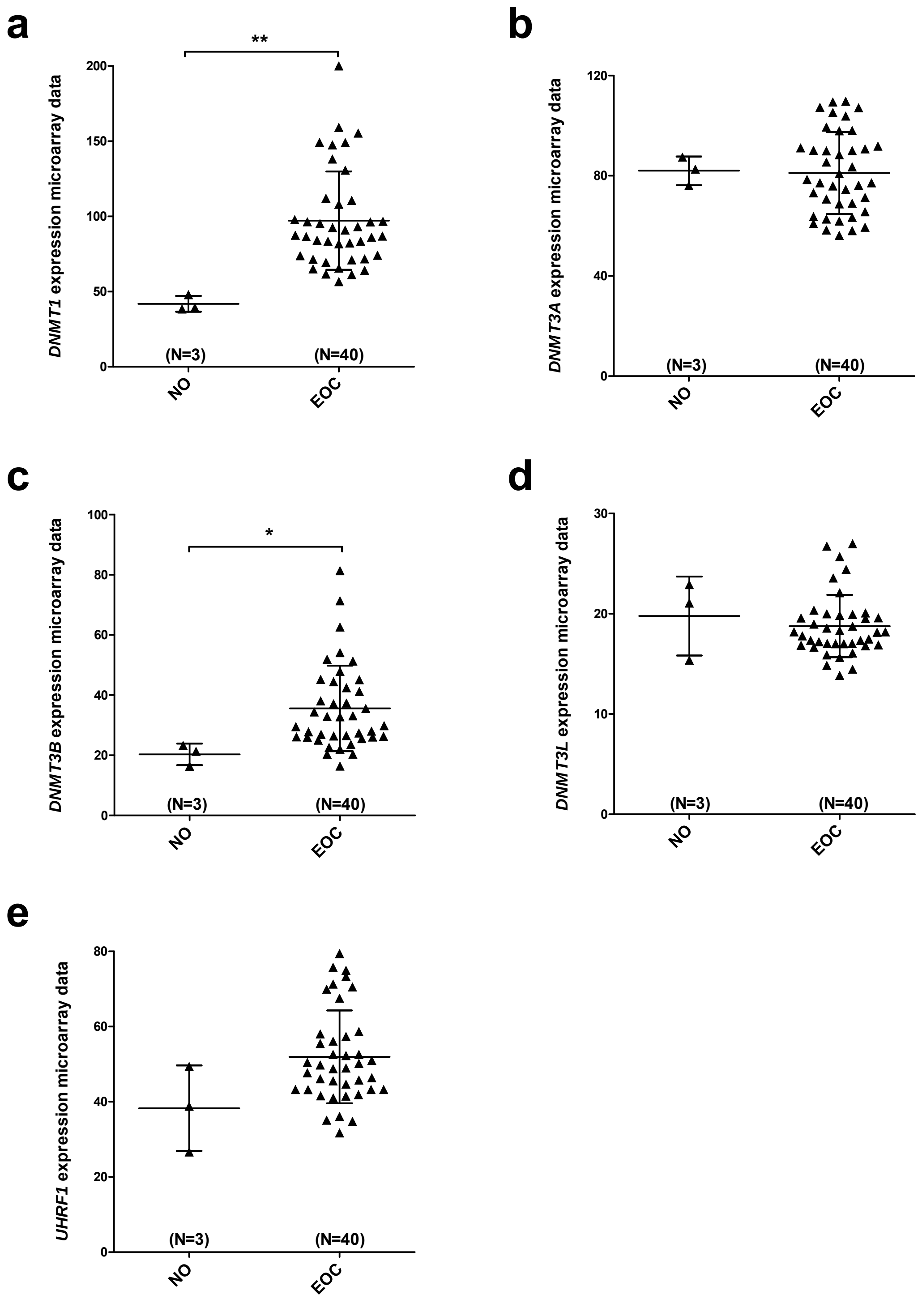


**Zhang et al. Supplementary Fig. 8.** ***DNMT1*, *DNMT3A*, *DNMT3B*, *DNMT3L*, and *UHRF1* expression in NO and EOC.** Gene expression was determined using standard Affymetrix RMA normalization. Mean +/− SD are plotted, and two-tailed Mann-Whitney test p-values are indicated (*p<0.05, **p<0.01).

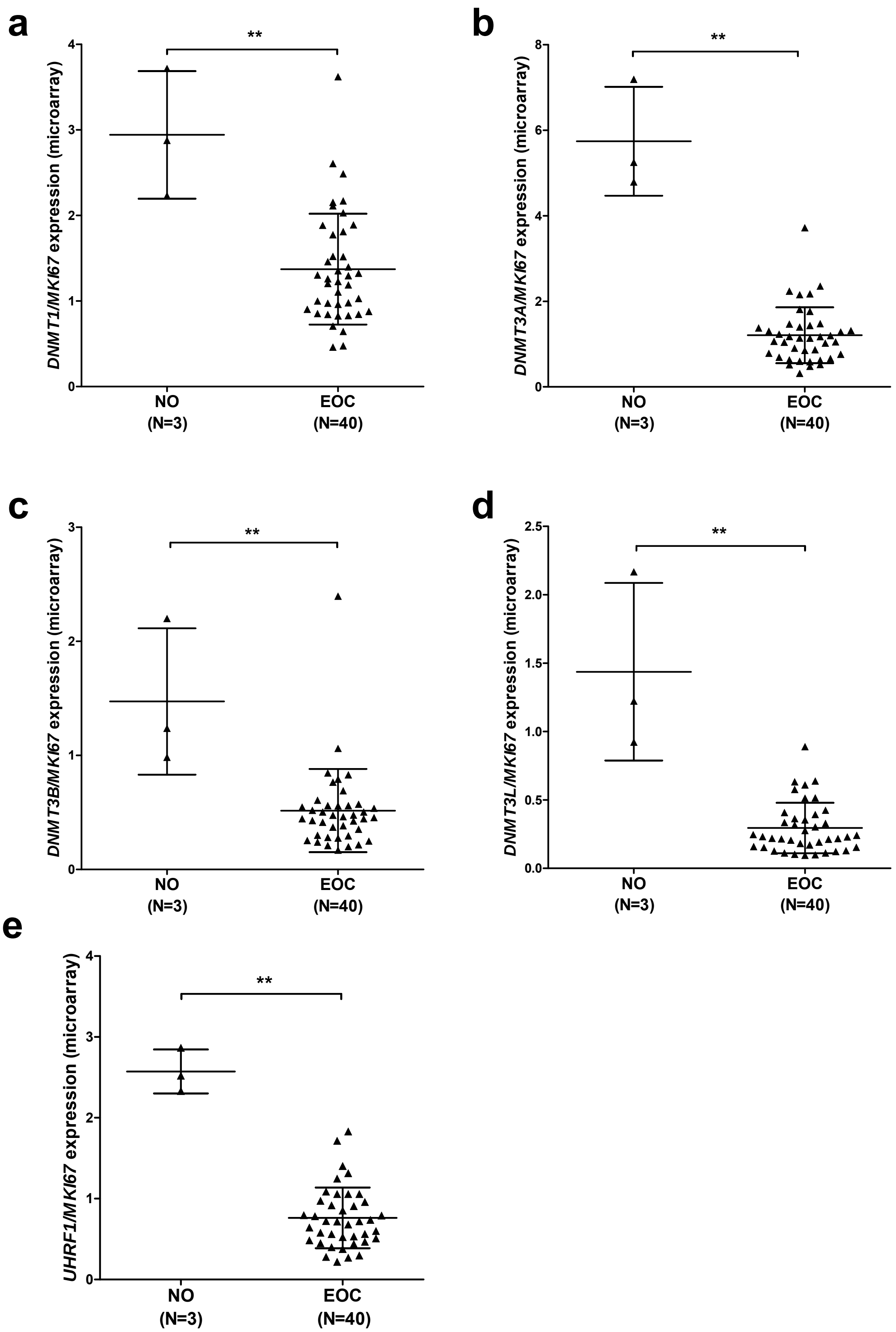


**Zhang et al. Supplementary Fig. 9. *DNMT1, DNMT3A, DNMT3B, DNMT3L, and UHRF1* expression, normalized to *MKI67*, in NO and EOC.** Gene expression was determined by Affymetrix microarray. Mean +/− SD are plotted, and two-tailed Mann-Whitney test p-values are indicated (*p<0.05, **p<0.01, ***p<0.001).

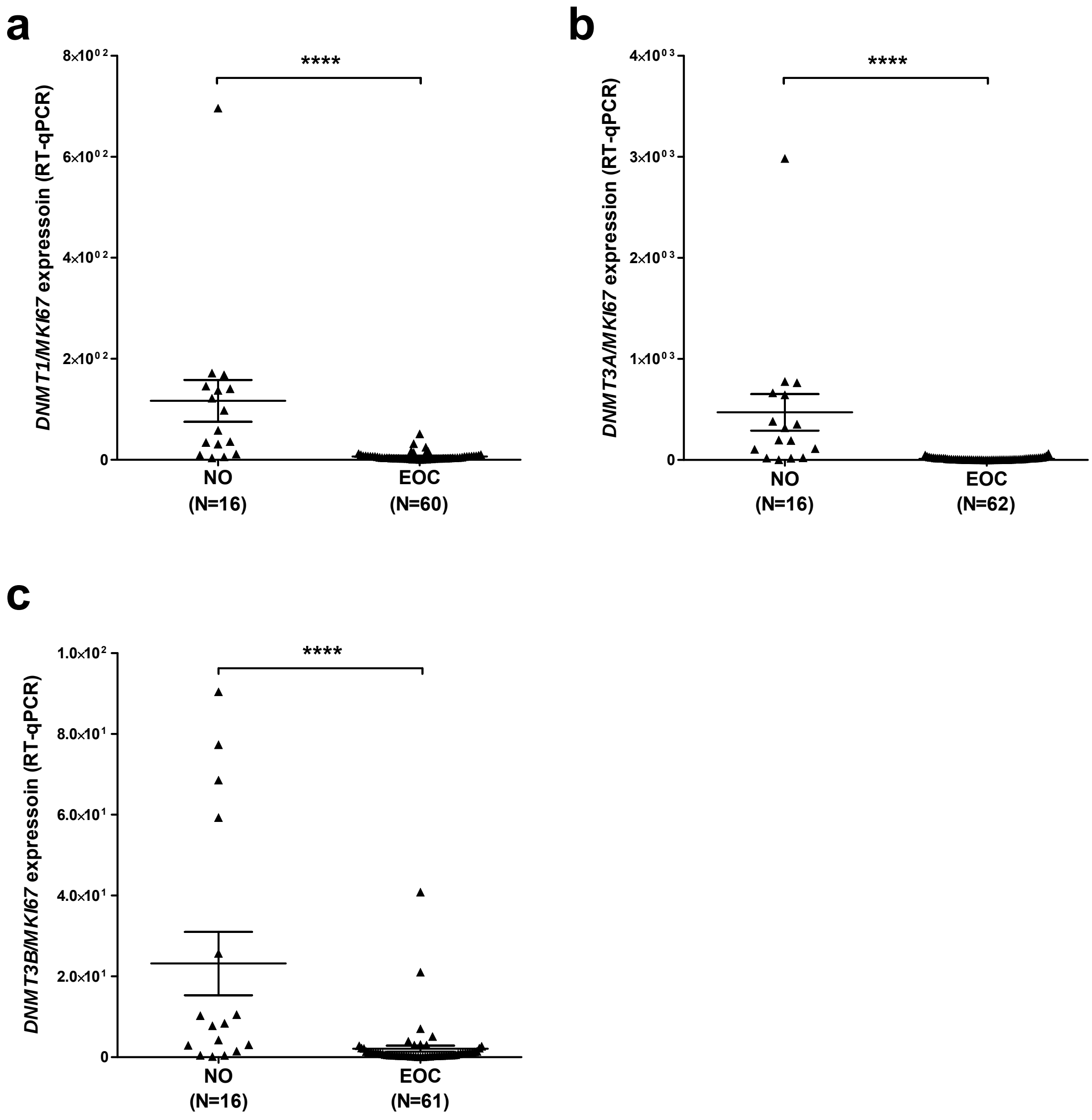


**Zhang et al. Supplementary Fig 10. *DNMT1, DNMT3A,* and *DNMT3B* expression, normalized to *MKI67*, in NO and EOC.** Gene expression was determined by RT-qPCR. Mean +/− SD are plotted, and two-tailed Mann-Whitney test p-values are indicated (****$p<0.0001$).

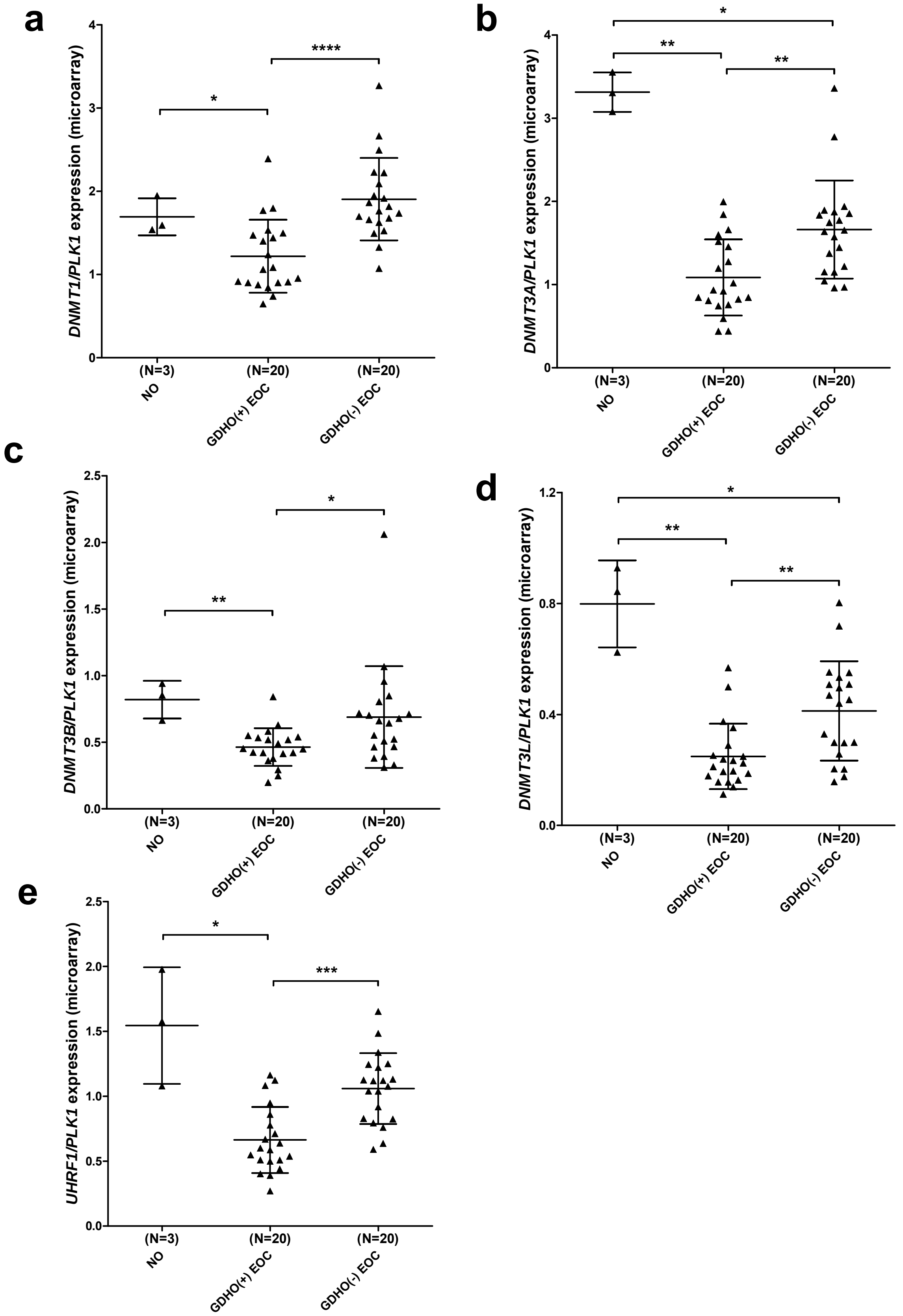


**Zhang et al. Supplementary Fig 11. *DNMT1, DNMT3A, DNMT3B, DNMT3L, and UHRF1* expression, normalized to *PLK1*, in NO, GDHO(+), and GDHO(–) EOC.** Gene expression was determined by Affymetrix microarray. Mean +/− SD are plotted, and two-tailed Mann-Whitney test p-values are indicated (*p<0.05, **p<0.01, ****p<0.0001).

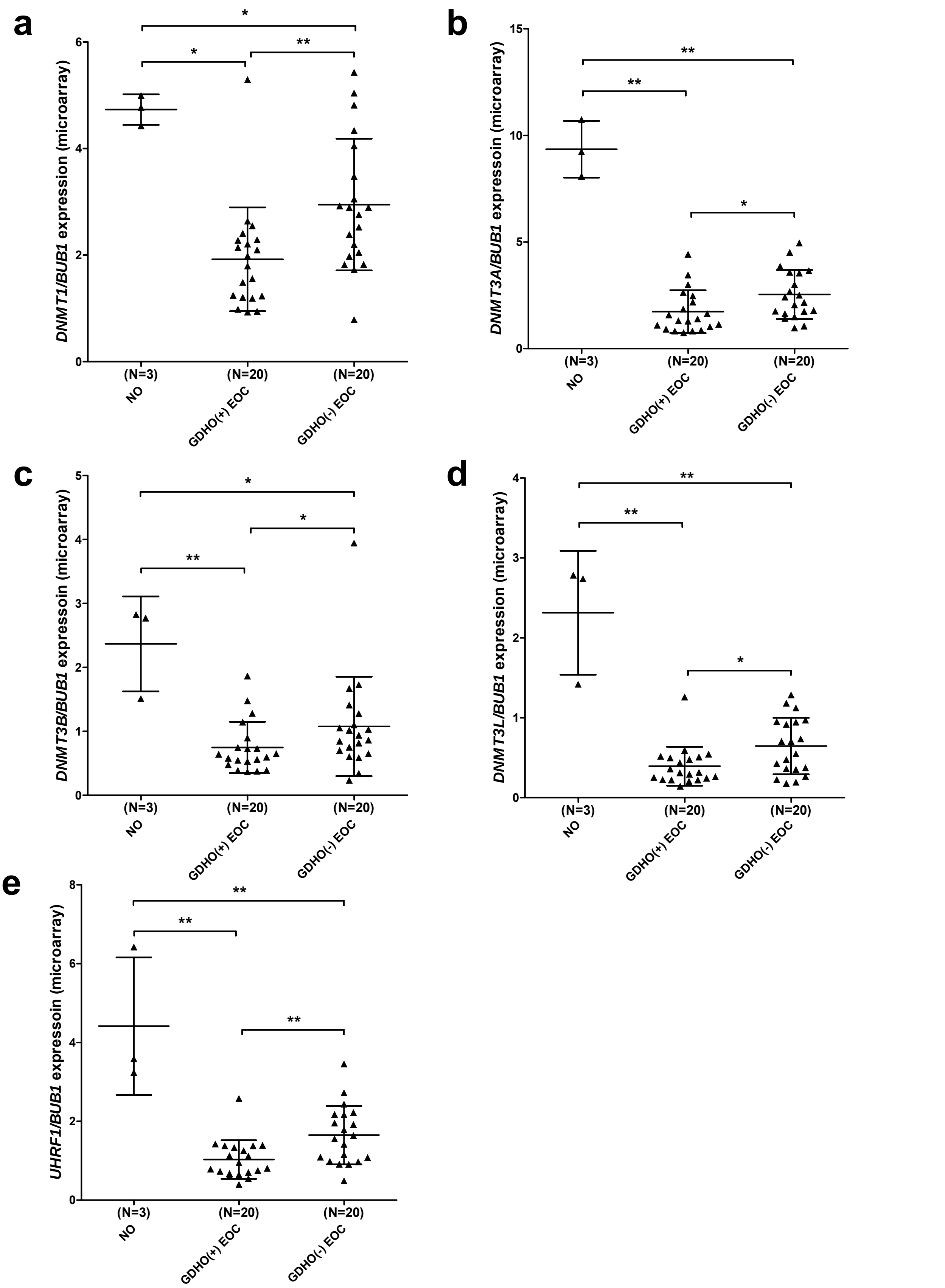


**Zhang et al. Supplementary Fig 12.** ***DNMT1, DNMT3A, DNMT3B, DNMT3L, and UHRF1*** **expression, normalized to** ***BUB1*****, in NO, GDHO(+), and GDHO(–) EOC.** Gene expression was determined by Affymetrix microarray. Mean +/− SD are plotted, and two-tailed Mann-Whitney test p-values are indicated (*p<0.05, **p<0.01).
