# Supplementary figures and images for: Global DNA Hypomethylation in Epithelial Ovarian Cancer: Passive Demethylation and Association with Genomic Instability
Source: Cancers (Basel). 2020 Mar 24;12(3):764. doi: 10.3390/cancers12030764 (PMC7140107; doi:10.3390/cancers12030764)

## Slide 1
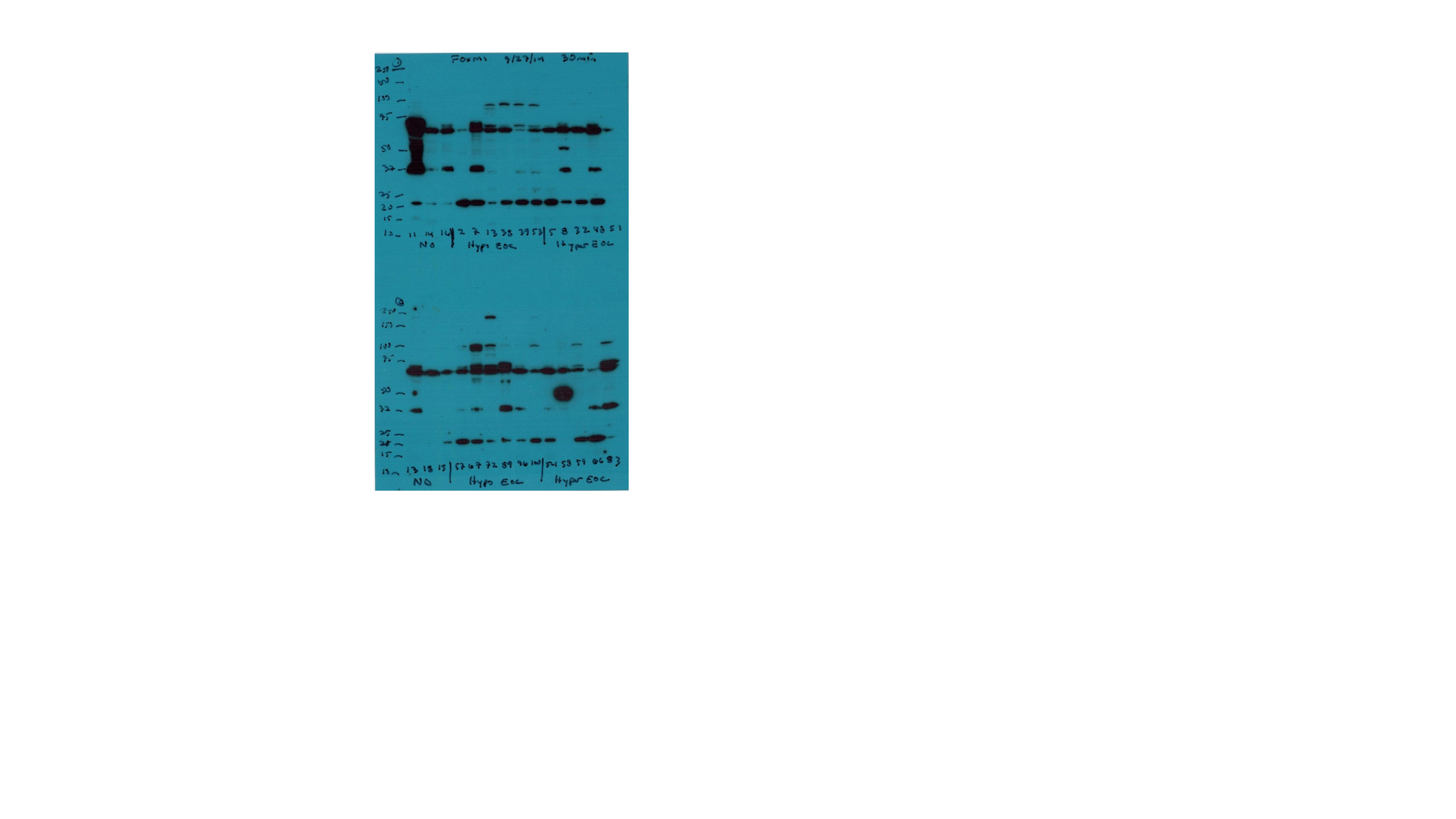

Supplement: Supplementary file 1 [file cancers-12-00764-s001.zip › FOXM1 Cancers uncropped Western blot.pptx]
